# Enrichment and characterization of human-associated mucin-degrading microbial consortia by sequential passage

**DOI:** 10.1093/femsec/fiae078

**Published:** 2024-05-24

**Authors:** Ashwana D Fricker, Tianming Yao, Stephen R Lindemann, Gilberto E Flores

**Affiliations:** Department of Biology, California State University, 18111 Nordhoff Street, Northridge, CA 91330, United States; Whistler Center for Carbohydrate Research, Department of Food Science, Purdue University, 745 Agriculture Mall Drive, West Lafayette, IN 47907, United States; Whistler Center for Carbohydrate Research, Department of Food Science, Purdue University, 745 Agriculture Mall Drive, West Lafayette, IN 47907, United States; Department of Biology, California State University, 18111 Nordhoff Street, Northridge, CA 91330, United States

**Keywords:** fermentation, gastrointestinal microbiome, microbial ecology, mucin

## Abstract

Mucin is a glycoprotein secreted throughout the mammalian gastrointestinal tract that can support endogenous microorganisms in the absence of complex polysaccharides. While several mucin-degrading bacteria have been identified, the interindividual differences in microbial communities capable of metabolizing this complex polymer are not well described. To determine whether community assembly on mucin is deterministic across individuals or whether taxonomically distinct but functionally similar mucin-degrading communities are selected across fecal inocula, we used a 10-day *in vitro* sequential batch culture fermentation from three human donors with mucin as the sole carbon source. For each donor, 16S rRNA gene amplicon sequencing was used to characterize microbial community succession, and the short-chain fatty acid profile was determined from the final community. All three communities reached a steady-state by day 7 in which the community composition stabilized. Taxonomic comparisons amongst communities revealed that one of the final communities had *Desulfovibrio*, another had *Akkermansia*, and all three shared other members, such as *Bacteroides*. Metabolic output differences were most notable for one of the donor’s communities, with significantly less production of acetate and propionate than the other two communities. These findings demonstrate the feasibility of developing stable mucin-degrading communities with shared and unique taxa. Furthermore, the mechanisms and efficiencies of mucin degradation across individuals are important for understanding how this community-level process impacts human health.

## Teaser

Mucin-degrading microbial communities were developed from three healthy humans, each with unique bacterial taxa and metabolic outputs.

## Introduction

Everyone has a personalized intestinal microbiome, which assists in normal digestion that is cultivated through their lifetime and tightly linked to both health and disease. The composition of each individual’s gut microbiome is highly dependent on nutrient intake (Mohammadkhah et al. 2018). Although individual microorganisms can be associated with some disease states, other diseases are associated with microbial community features, examples include organisms that are efficient at dietary energy extraction associated with obesity (Liu et al. 2021) and organisms that contain sulfur metabolic machinery associated with colorectal cancer (Wolf et al. 2022). These microorganisms may affect host health through the production of metabolites that interact with local and distal tissues (Parada Venegas et al. 2019). For example, the production of short-chain fatty acids (SCFA) by fermentative gut bacteria has been implicated in protection of the epithelium, immune regulation, and epithelial cell proliferation and differentiation (Bilotta and Cong 2019). The mechanisms that maintain and bolster these functions of the microbiome are poorly understood but of great interest because of their potential to modulate health.

Microorganisms within the intestinal tract require basic resources including carbon and energy for growth and survival, which they derive primarily from the host diet (Leeming et al. 2019). Dietary fibers, as one example, compose a broad array of polysaccharides exclusively metabolized by diverse members of the gut microbiota. Recent evidence indicates that dietary fibers with more complex chemical structures necessitate a diverse array of enzymes for digestion often produced by specialist microorganisms (Lindemann 2020). These specialists enable a myriad of microbial interactions as they liberate smaller sugar subunits and produce fermentation products that are both used by other microbiota members and by host cells (Koropatkin et al. 2012). Comparatively simple fibers, such as fructooligosaccharides and type II resistant starch, require fewer enzymes for degradation, making these polysaccharides more widely accessible to many gut organisms (Cantu-Jungles et al. 2021). Many individual microorganisms within complex microbial communities also require exogenous forms of vitamins and amino acids due to underlying auxotrophies (Yu et al. 2022). While in the native gastrointestinal tract, these essential nutrients may be derived from the host diet; in the cultivation of fecal microbial consortia, supplementation drastically affects final community outcomes (Zengler and Zaramela 2018, Yao et al. 2020). Auxotrophy for amino acids *in vitro* can be alleviated through addition of free amino acids, peptides, or protein supplements. However, the ability of an organism to scavenge amino acids varies; only some organisms produce exopeptidases capable of breaking down complex proteins into smaller fragments, such as dipeptides or free amino acids, which are then transported into the cell (Zhang et al. 2022). Therefore, variability in host diets leads to a difference in dietary fibers and amino acid sources consumed, ultimately conferring advantages to specific sets of microorganisms capable of breaking the discrete bond types and helping to shape the uniqueness of each microbiome.

Despite this individuality in diet, healthy humans secrete mucins, a family of glycoproteins decorated with O-linked carbohydrates in various configurations produced by specific epithelial cells in the intestinal, respiratory, and genitourinary tracts. Although primarily known for their role as a barrier against invasion, the high polysaccharide content of mucins can potentially serve as a rich and reliable nutrient source for microorganisms (Tailford et al. 2015). Mucin structure is complex, fundamentally containing a serine–threonine–proline-rich protein backbone, an *N*-acetylgalactosamine (GalNAc) O-linked to this protein, an array of sugars linked to the GalNAc, and a final terminal unit (Corfield 2015). The breadth in structures lies in the sugar array, which can comprise α-(1–3)- or β-(1–3)-linked galactose, or a β-(1–6)-, β-(1–3)-, α-(1–6)-linked *N*-acetylglucosamine (GlcNAc). Furthermore, these structures can be capped by a sulfate group, fucose, or *N*-acetylneuraminic acid (sialic acid; NeuAc). Given this diversity of carbohydrate chemical linkages and a limited number of amino acids, only a subset of gut microorganisms possess the functional capacity to hydrolyze and utilize mucins for growth (Glover et al. 2022).

Some intestinal microorganisms, such as the nutritional generalist *Bacteroides thetaiotaomicron*, can switch between fermentation of dietary polysaccharides and mucin glycans (Ravcheev et al. 2013). During fermentation of either dietary fibers or mucin, byproducts, including the SCFA acetate, are produced (Mahowald et al. 2009). These metabolites stimulate mucin production by goblet cells in the gut, resulting in an intact mucus barrier between human and bacterial cells along the intestines (Willemsen et al. 2003, Adamberg et al. 2018). In fact, there appears to be a direct connection between the abundance of dietary fiber in the diet and colonic mucin thickness (Brownlee et al. 2003, Hedemann et al. 2009, Earle et al. 2015, Desai et al. 2016), likely mediated by the microbiota.

A smaller subset of organisms in the gastrointestinal tract, like the mucin-degrading specialist *Akkermansia muciniphila*, appear to depend on direct mucin fermentation for growth (Derrien et al. 2004). There are many carbohydrate-active enzymes (CAZymes) responsible for metabolizing complex mucin glycans, where each enzyme (e.g. glycoside hydrolase—GH) is responsible for hydrolysis of a specific glycosidic bond, liberating specific sugar moieties. Although it is possible that *Akkermansia*, a specialist that retains a full suite of mucin-degrading CAZymes, is capable of filling this mucin-degrading niche and outcompeting other organisms for this resource, the incidence rate is only 50%–80% of the human population (Geerlings et al. 2018). Given that much of the established abundances of microorganisms in the human gut are confined by available technology, including *in vivo* sampling, DNA extraction methods, primer choice, and detection limits (Brandt and Albertsen 2018, Abellan-Schneyder et al. 2021), *Akkermansia* may in fact be present in all gut communities. Alternatively, it is possible that gut microbial communities divide the metabolic labor in which partial hydrolysis of mucin releases mono- or disaccharides that can then be fermented by other organisms (Bunesova et al. 2018). In this second scenario, the activities of secondary consumer microbiota would be supported by the intestinal mucin and further stimulate mucin production, thereby limiting pathogen invasion. To determine whether mucin metabolism would be monopolized by a single member or support a complex community, we sought to characterize the indigenous microbial communities capable of growth on mucin across multiple individuals.

Here, we use sequential transfer in media with mucin as the sole carbon source to investigate the diversity, composition, and taxonomy of native gut microbial consortia from three human donors. We expected that each consortium would reach a steady state and, while some taxa may be shared across donors, each community would be distinct. Additionally, within each donor, we expected replicate lineages to be similar due to strong, nutritional selection imposed by the mucin glycoprotein. However, the amino acid sources provided will influence overall community composition within each individual. By using a top-down approach, we identify a core and unique set of microorganisms involved in mucin metabolism with distinct SCFA production profiles.

## Methods

### Medium

Fermentation medium was based on Yao et al. (2020), with modifications. Briefly, our fermentation medium contained 24 µM Na_2_HPO_4_, 15.6 µM NaH_2_PO_4_, 8 mM NaCl, 6 mM KCl, 6.7 mM NH_4_Cl, 0.7 mM Na_2_SO_4_, 1 ug/ml resazurin, 3.2 mM urea, 1.4 mM cysteine-HCl, 0.5 mM CaCl_2_-2H_2_O, 0.5 mM MgCl_2_-6H_2_O, and trace minerals solution. Trace minerals solution is based on Ferguson and Mah (1983), where the final composition in fermentation medium consisted of 1.3 µM Na_2_-EDTA-2H_2_O, 0.6 µM CoCl_2_ 6H_2_O, 0.5 µM MnCl_2_-4H_2_O, 0.36 µM FeSO_4_-7H_2_O, 0.7 µM ZnCl_2_, 0.17 µM AlCl_3_-6H_2_O, 0.09 µM Na_2_WO_4_-2H_2_O, 0.12 µM CuCl_2_-2H_2_O, 0.076 µM NiSO_4_-6H_2_O, 0.077 µM H_2_SeO_3_, 0.16 µM H_3_BO_3_, and 0.04 µM NaMoO_4_-2H_2_O. To alleviate amino acid auxotrophies, either 10 µM equimolar amino acid mix or 0.1 mg/ml tryptone (Oxoid) was added. Tryptone concentration was normalized to the amino acid mix based on the percentage of l-cysteine as published by USBiological Life Sciences (2024). Compositional differences between the amino acid sources are indicated in Supplemental Table S1.

#### Soluble mucin preparation

Preparation of soluble mucin was based on Kirmiz et al. (2020) and added to a final concentration of 0.5%. Briefly, soluble porcine gastric mucin type III (Sigma–Aldrich, USA) was prepared by autoclaving a 5% (w/v) solution in 0.01 M phosphate buffer (6.5 mM KH_2_PO_4_ and 3.5 mM K_2_HPO_4_), dialyzing with a 12–14-kDa membrane (Spectra/Por 4; Spectrum Laboratories, Rancho Dominguez, CA) overnight in 10 dialysis volumes of 0.01 M phosphate buffer, centrifuging for 30 min at 30 000 rcf, and filtering through a 0.45-µm syringe filter followed by a 0.2-µm syringe filter (Whatman GE Healthcare Life Sciences, Chicago, IL). Sterile soluble mucin was then added to the fermentation medium at a final concentration of 0.5%.

#### Mixing of mucin solution and fermentation medium

Briefly, concentrated fermentation medium (2X) was aerobically prepared and diluted to a working concentration with soluble mucin and sterile water. Prior to transfer to Balch tubes, buffer–mucin mix was stored loosely covered in the anaerobic chamber (85% N_2_, 10% CO_2_, and 5% H_2_) for 48 h to outgas. Each tube received 5 ml fermentation medium except the initial three tubes which each received 4 ml. Fermentation medium containing mucin was aseptically distributed into Balch tubes in a 10% CO_2_, 5% H_2_, and 85% N_2_ atmosphere before sealing with butyl rubber stoppers. After preparation, tubes were stored at 37°C prior to inoculation to detect any contamination. Immediately before inoculation, 0.05 ml ATCC vitamin mix (final 1% v/v, manufacturer’s number: ATCC MD-VS, Hampton, NH) was aseptically added to each tube. An additional three tubes were maintained for the full duration of the experiment at 37°C without inoculation as negative controls.

### Inoculum preparation

Approximately 4 ± 1.5 g of fecal material was collected from three healthy donors representing two female and one male donor ranging from 20 to 45 years of age. Donors had not received any antibiotic treatment within 3 months and were generally in good health. The protocol for collecting human feces was approved by the Institutional Review Board of California State University, Northridge (#1516–146-f). Donors were asked to collect two Hershey’s kiss size samples (∼4.2 g each) into separate preweighed conical vials using sterile wooden spatulas. Samples were maintained on blue ice during transfer following best practices for retaining viable organisms (Owens et al. 2013) and received within 3 hours of evacuation. Fecal material was diluted 1:4 w/v with 1X fermentation medium containing mucin as described above. Tubes were vortexed for 20 s, returned to ice for up to 5 min, and vortexed again for 20 s. The fecal slurry was then poured over 4-ply sterile cheesecloth, and the flow through was collected with a syringe as previously described (Yao et al. 2020, Romero Marcia et al. 2021, Tuncil et al. 2017, 2018). To represent day 0 (the initial inoculum), 500 µl of this fecal slurry was centrifuged at 10 000 × *g* for 5 min and the cell pellet stored at −20°C until thawing for DNA extraction. Each tube containing 4 ml of fermentation medium then received 1 ml of fecal slurry totaling a 1:20 fecal dilution. The initial inoculation tubes received an additional 50 µl of 0.25 g/ml cysteine to reduce the initial inoculum for a final total cysteine concentration of 2.4 mM. Tubes were placed in a static 37°C incubator inside of an anaerobic Coy chamber (10% CO_2_, 5% H_2_, and 85% N_2_ atmosphere) for 23–25 h before sample collection and transfer as described below.

### *In vitro* sequential fecal fermentation

The sequential cultivation experiment was continued for 10 consecutive days, and each donor was cultured in triplicate without intermixing the replicates, resulting in three independent lineages for each donor and amino acid source (Yao et al. 2020). Daily, immediately prior to inoculation, 50 µl of ATCC vitamins (final 1% v/v, manufacturer’s number: ATCC MD-VS, Hampton, NH) was added to each 5 ml fermentation medium. Due to settling of microorganisms from static incubation, tubes were gently inverted 5x prior to sampling. Subsequently, as previously described (Yao et al. 2020), 50 µl of the incubated culture was transferred to the corresponding tube containing 5 ml fermentation medium and incubated in the Coy chamber as indicated above.

Daily, 500 µl of each incubated culture was collected and centrifuged at 10000 × *g* for 5 min. The cell pellet was stored at −20°C until thawing for DNA extraction. On the final day, after centrifugation, the supernatant was transferred to a separate microfuge tube and stored at −20°C until thawing for SCFA detection as indicated below.

### DNA extraction and sequencing

DNA was extracted from cell pellets collected above using the Zymo DNA Microprep Kit #D4301 (Zymo Research Corporation, USA), with the following modifications. For the initial inoculum, 20% of the resuspended culture was processed to prevent overloading the kit. After resuspension and transfer to the BeadBashing lysis tubes, samples were placed on a horizontal vortex adapter at max speed for 10 min. DNA concentrations were read on a Qubit 2.0 fluorometer with the high-sensitivity kit, following manufacturer’s protocol. A volume of 1 µl of each eluted sample was used in polymerase chain reaction amplification of the variable region 4 of bacterial and archaeal 16S ribosomal RNA genes with barcoding primer set 515/806 based on the original Earth Microbiome Project protocol (Caporaso et al. 2011, Earth Microbiome Project 2024). Primers used were 515F (Parada et al. 2016)—GTGYCAGCMGCCGCGGTAA and 806R—GGACTACHVGGGTWTCTAAT with barcoding on the 806R primer. PCR mixture conditions and thermal cycling steps are previously described (Herman et al. 2020). Triplicate PCR reactions for each sample were combined, quantified with Quant-iT PicoGreen (Invitrogen) on a SpectraMax3 (Molecular Devices, USA), and pooled at equimolar concentrations before single-tube cleaning with the Invitrogen Gel and PCR Clean-up Kit (Invitrogen) and sequencing using an Illumina MiSeq (v2, 2 × 150 bp) instrument (Illumina, USA).

### DNA sequence analysis

Sequences were analyzed following the QIIME-2 Atacama Desert pipeline (Qiime2Docs), briefly described here. Sequences were denoised, dereplicated, and chimeras were removed with Dada-2 (Callahan et al. 2016) with the following parameters: –p-trim-left 5 –p-trunc-len 150. Amplicon sequence variants (ASVs) were classified using sklearn and the silva 138.99 database (Quast et al. 2012). PCR blanks were used to remove contaminating ASVs following the decontamR pipeline (Davis et al. 2018) using a prevalence-based strategy. Given that the experiment occurred with triplicate lineages, after combining the two datasets, ASVs that were present in less than two of the total samples were removed. Subsequently, samples with fewer than 5000 reads, representing the extraction blanks, were removed. This resulted in a total of 192 samples for downstream analysis, which included six samples rerun to compare differences across sequencing runs. QIIME-2 was used to calculate various α-diversity (total ASV counts and Shannon diversity index) and β-diversity (Bray–Curtis) metrics.

### Determination of fatty acids

Final consortia cultures at day 10 were analyzed for SCFAs of acetate, propionate, butyrate, and branched-chain fatty acids (BCFAs) of isobutyrate and isovalerate. Samples were measured on a GC-FID system (GC-2030, Nexis, Shimadzu Corporation, Kyoto, Japan). Cultures were centrifuged (10 000 × *g*) for 5 min to remove cell debris, mixed 4:1 with an internal standard mixture containing 4-methylvaleric acid, phosphoric acid, and copper sulfate pentahydrate, and supernatants were transferred to 2 ml screw-thread autosampler vials. Volatile compounds were quantified on a GC-FID with a wax column (RESTEK Stabilwax-DA #11023, Bellefonte, PA). Helium was used as a carrier gas for the column at a flow of 1.41 ml/min. Samples were injected in a split mode (5 ul injection volume, 1:5 split ratio) under a linear velocity of 35 cm/s at 230°C. The oven temperature was set as below: initial temperature at 40°C for 2 min, following a ramp to 130°C in 3 min, and then another ramp to 195°C in 6.5 min, holding at 195°C for 4 min.

### pH and OD testing

On the final day of sampling, tubes were removed from the anaerobic chamber and opened aerobically. Cultures were transferred to a 15-ml conical vial and 100 µl of the culture was used to determine OD_600nm_ on a Biophotometer plus spectrophotometer (Eppendorf, Germany). Cultures with an OD_600nm_ >1.0 were diluted with sterile water and reread. The pH was tested on the remaining volume using an accumet pH meter (Fisher Scientific, USA).

### Statistical analysis

For pH, OD_600nm_, SCFA, and alpha diversity analysis, statistically significant differences were calculated by Tukey’s honest significance multiple comparisons test using R 4.1.1 (R Foundation for Statistical Computing, Vienna, Austria). Comparisons were considered significant if corrected *P*-values were less than .05. For beta diversity analysis, dissimilarity matrices generated in Qiime-2 were used to calculate statistically significant differences with ADONIS from the vegan package in R 4.1.1. Comparisons were considered significant if the *P*-value of the *F* statistic was less than .01. Symbol style for figures: nonsignificant (ns), 0.05 (*), 0.01 (**), 0.001(***), and <0.0001(^****^).

## Results

To better understand the ecology of mucin degradation as a community level process by the human gut microbiota, we performed 10-day sequential fecal enrichment from three human donors in triplicate, using purified porcine gastric mucin as the sole carbon and energy source. As a secondary variable and to determine the impact of amino acid source on microbial community composition, we used two amino acid sources during community development in parallel enrichments for each donor. For each day, cultures were transferred at a high (10^−2^) dilution rate and cell pellets were collected to monitor community development using 16S rRNA gene amplicon sequencing. On the final day, the OD_600nm_ and pH of cultures was tested, and the spent culture supernatant was collected and used to determine SCFA production.

### Amino acid source does not govern growth or metabolic outputs

**Figure 1.**
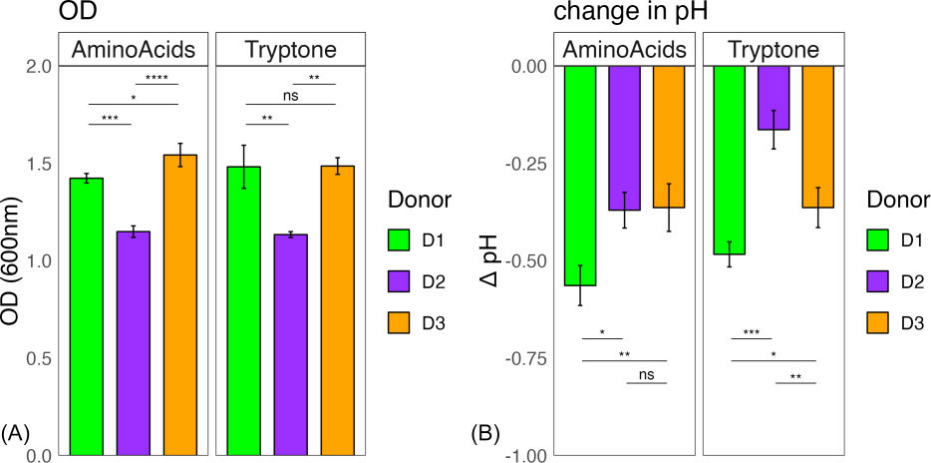
Final (day 10) optical density and pH measurements of microbial consortia from three human donors established with mucin as the sole carbon source supplemented with different amino acid sources. Each community grew to different final optical densities (A) and produced different levels of acid (B) after 10 days of incubation. Averages of three lineages from each final donor community ((Donor 1, D1; Donor 2, D2; and Donor 3, D3) are displayed with error bars representing standard deviation as calculated in R. Statistically significant differences are calculated by Tukey’s multiple comparisons test with *P* < .05. Symbol style: nonsignificant (ns), 0.05 (*), 0.01 (**), 0.001(***), and <0.0001(^****^).

In cultures from all three donors, robust growth was evident by changes in media turbidity after each day of incubation, regardless of amino acid treatment. On the final day of growth, all communities grew to OD_600nm_ >1 and there were no significant differences in OD_600nm_ within a donor’s communities on either amino acid source (Supplemental Fig. S1). However, across donors, communities from donors one and three grew to significantly higher OD_600nm_ (∼1.4–1.6) than donor two (∼1.1) suggesting difference in growth yield (Fig. 1A). Similarly, drops in pH were also generally greater for communities from donors one and three, except for on the amino acid mixture where donor two and three communities both dropped ∼0.3 pH units (Fig. 1B). Interestingly, communities from donor two had a significantly different drop in pH between the two amino acid sources, with the culture growing on mixed amino acids dropping to pH 6.1 and the culture grown on tryptone to 6.3 (Supplemental Fig. S1). This potentially suggests ammonification of the medium from degradation of the more abundant proteins in the community cultivated on tryptone.

### SCFA output is unique to each community

**Figure 2.**
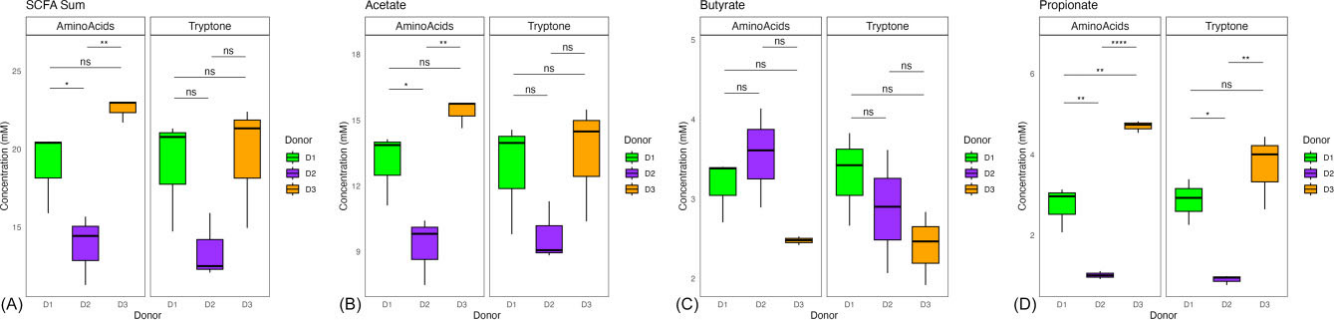
SCFA profiles of microbial consortia from three human donors established with mucin as the sole carbon source after 10 days of sequential transfer. Mucin-degrading consortia from donors one and three produced the highest total concentration of SCFAs driven by acetate and propionate, whereas the donor two consortium produced lower concentrations, irrespective of amino acid source. Mean, first, and third quartiles of total SCFAs (A), acetate (B), propionate (C), and butyrate (D) for each final donor community (Donor 1, D1; Donor 2, D2; and Donor 3, D3) are represented. Statistically significant differences are calculated by Tukey’s multiple comparisons test. Symbol style: nonsignificant (ns), 0.05 (*), 0.01 (**), 0.001(***), and <0.0001(^****^).

SCFAs are main metabolic byproducts of gut microbial fermentation of complex carbohydrates. Therefore, to determine the metabolic output of each community during mucin fermentation, the SCFA profile was assayed on day 10 of sample collection (Fig. 2). Overall, microbial consortia from donors one and three produced the highest total concentration of SCFAs (∼20 mM, sum of acetate, butyrate, and propionate) whereas the donor two community produced lower concentrations (14 mM), irrespective of amino acid source. These differences were largely driven by acetate and propionate, where cultures from donors one and three produced more than donor two. However, differences of specific SCFAs generated by each donor’s communities were dependent on amino acid source as discussed below.

When cultivated on tryptone, communities from donors one and three produced equal amounts of propionate (3–4 mM), whereas the donor two consortium produced significantly less (0.5 mM). In contrast, there was no significant difference in the production of acetate (9–14 mM) or butyrate (2.5–3.5 mM) across the donor communities, when grown on this amino acid source. Although the same general trend of SCFA production was observed in cultures grown with the amino acid mix, the differences between communities was more pronounced. In these communities, donor three microbiota produced the greatest amounts of propionate (5 mM), followed by donor one microbiota (2.5 mM), with donor two’s microbiota generating low concentrations (0.5 mM). Concentrations of acetate produced by both donor one and donor three communities were similar (13–15 mM), with donor two’s producing lower concentrations (9 mM). The production of butyrate in amino acid mix-consuming communities mirrored those cultivated on tryptone, where there was no significant difference in the amount of butyrate produced (2.5–3.5 mM). Furthermore, we detected trace amounts of the BCFAs, isovalerate, and isobutyrate (Supplemental Fig. S2). These minor BCFAs reflected the patterns observed for SCFAs but had very low concentrations. Overall, these data coupled with the OD_600nm_ results, suggest mucin fermentation by microorganisms from all three donors.

### rRNA amplicon sequence quality and taxonomy

Across two MiSeq Illumina sequencing runs, a total of 13 120 305 sequences were obtained. Each run was independently denoised, dereplicated, and chimeras were removed. Amplification blanks were used to remove contaminating sequences, where 66 ASVs from the first sequencing run and 9 ASVs from the second were removed. After removal of sequences present only in a single sample and extraction blanks, the final number of sequences analyzed constituted 92.9% of the original sequence total (12 184 363 sequences). To determine whether sequencing run bias would influence the results, six samples representing the initial inoculum and mid-to-end timepoints from both donors one and two were sequenced on both sequencing runs and community composition was compared (Supplemental Table S2). The differences in β-diversity (Bray–Curtis dissimilarity) across samples sequenced on the first and second run were not significantly different as determined by adonis analysis (*R*^2^ = 0.003, *P* = .95).

### Final community composition and diversity is dependent on initial inoculum

**Figure 3.**
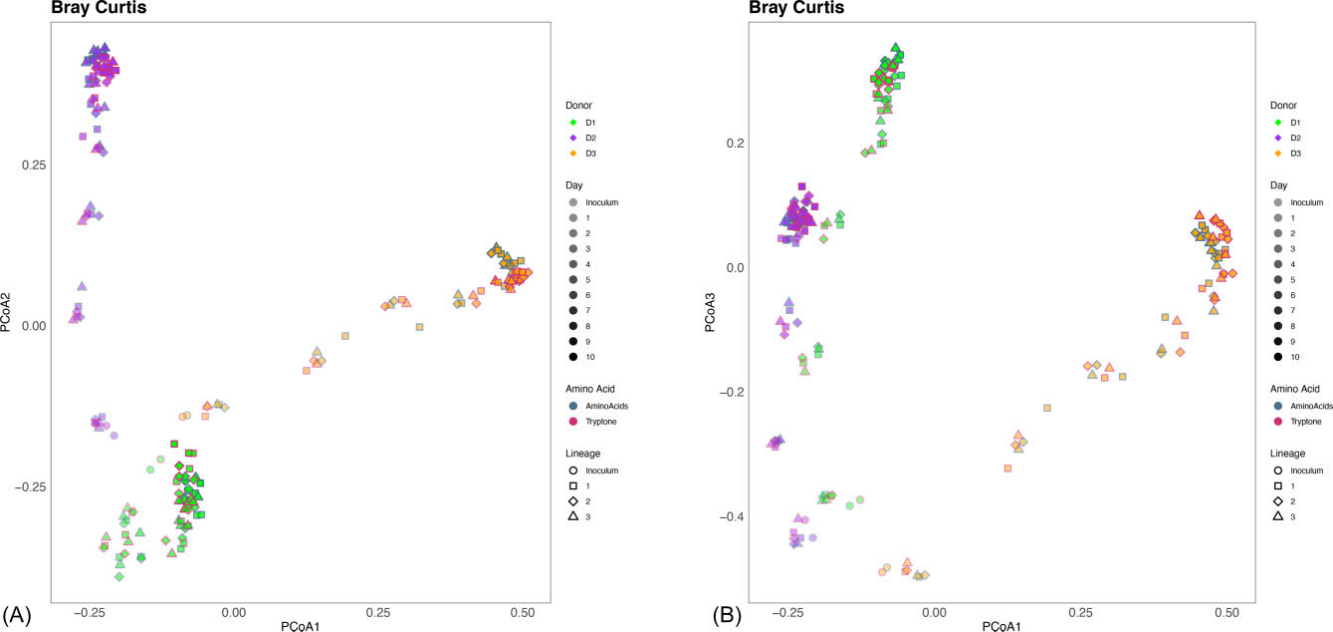
Principal coordinates of analysis plots of Bray–Curtis dissimilarity between communities for each donor over 10 days with supplementation of either amino acids or tryptone. Donors are represented by fill color, days by hue of the dominant color, amino acid supplementation by border color, and lineage by shape. Principle coordinates 1 and 2 (A) and 1 and 3 (B) indicate that the community from each donor follows a distinct trajectory.

Six mucin-degrading communities were established from three human donors to test whether mucin deterministically selects for specific community compositions both across and within individual human donors. To determine community composition and diversity through time, standard ecological diversity metrics were calculated based on profiling of the V4 region of the 16S rRNA gene sequence. As expected, initial fecal communities were compositionally unique from one another although we could not statistically test this since only a single sample was collected from each donor. Subsequently, since we developed triplicate lineages for each amino acid source, we could statistically test for differences across and within donors through time. Across donors, each community was compositionally distinct throughout development regardless of nitrogen source (Supplemental Table S3). Within each donor, microbial community development followed the same trajectory across the replicate lineages regardless of amino acid source (Fig. 3), suggesting that community composition is governed more by mucin than by amino acid source under these conditions. These results also show that community assembly within a donor is replicable under high dilution pressure with mucin.

To determine whether communities were persistent throughout time, we first calculated the Bray–Curtis dissimilarity and compared differences between consecutive days within each donor (Supplemental Fig. S3). Given that there were no significant differences within each donor’s communities between amino acid sources, we then grouped amino acid sources for each day to identify community plateau for each donor (Supplemental Table S3). Comparing the communities at each day to the previous day indicates that the differences between communities after day 6 or 7 was not significant (Supplemental Table S4), suggesting communities had stabilized by this point.

**Figure 4.**
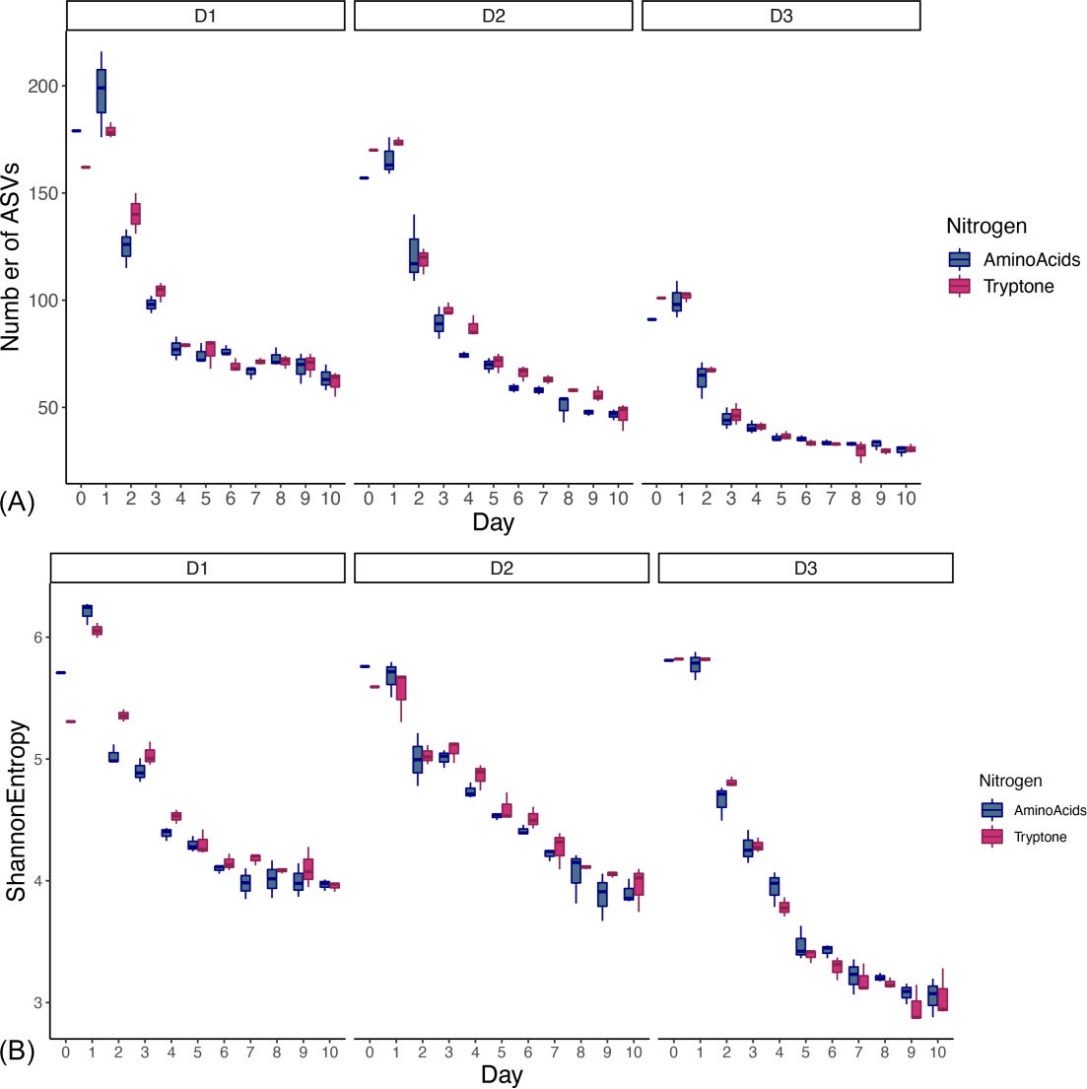
Bacterial diversity at each passage for all three donors (Donor 1, D1; Donor 2, D2; and Donor 3, D3) when supplemented with amino acids provided as an equimolar mix ("AminoAcids") or peptides ("Tryptone"). Species richness as measured by the number of ASVs (A) and evenness as measured by Shannon entropy (B) plateaued for all three communities. Mean, first, and third quartiles are represented with fill color indicating amino acid source. Statistically significant differences at day 10 are calculated by Tukey’s multiple comparisons test. Symbol style: nonsignificant (ns), 0.05 (*), 0.01 (**), 0.001(***), and <0.0001(^****^).

From an alpha diversity perspective, similarities and differences within and across donor communities were observed depending on diversity metric (Supplemental Table S5). For example, by approximately day 5, richness (ASV count) of each community plateaued (Fig. 4, Supplemental Table S6). In contrast, the Shannon diversity metric, which accounts for both richness and evenness, suggests that donor one communities leveled off earlier than donors two and three (Fig. 4, Supplemental Table S7). Interestingly, the amino acid source affected community richness for Donor 1 on days 2 and 4, and community evenness for Donor 2 on days 4, 6, 7, and 9, but not for the Donor 3 community (Supplemental Tables S5, S8, and  S9). Overall, these results demonstrate that although the timing differs across donors, all six communities reached a steady-state.

Each donor started and ended with a different final number of ASVs (Fig. 4), where donors one and two started with the greatest diversity at 171 ± 8 ASVs and 164 ± 6 ASVs, respectively. These communities plateaued to communities with 63 ± 7 (donor one) and 48 ± 4 (donor two) ASVs. Interestingly, donor three had much lower initial diversity, starting at about half the initial number of ASVs (96 ± 5) compared to donors one and two, and reached a correspondingly lower number of 31 ± 2 ASVs. However, the % loss was approximately equal across donor samples, where each final community represented ∼30% of the initial community (Donor 1, 35.5%; Donor 2, 29.7%; and Donor 3, 31.8% Supplemental Fig. S4). Furthermore, the point at which the loss in ASVs plateaued differed among the communities; communities belonging to donors one and two took 4 and 5 days, respectively, to reach this plateau, whereas donor three communities took 3 days (Supplemental Table S10). Taken together, these data suggest that mucin can sustain diverse fermenting consortia over sequential dilutions in a manner related to the initial community structure and diversity.

**Figure 5.**
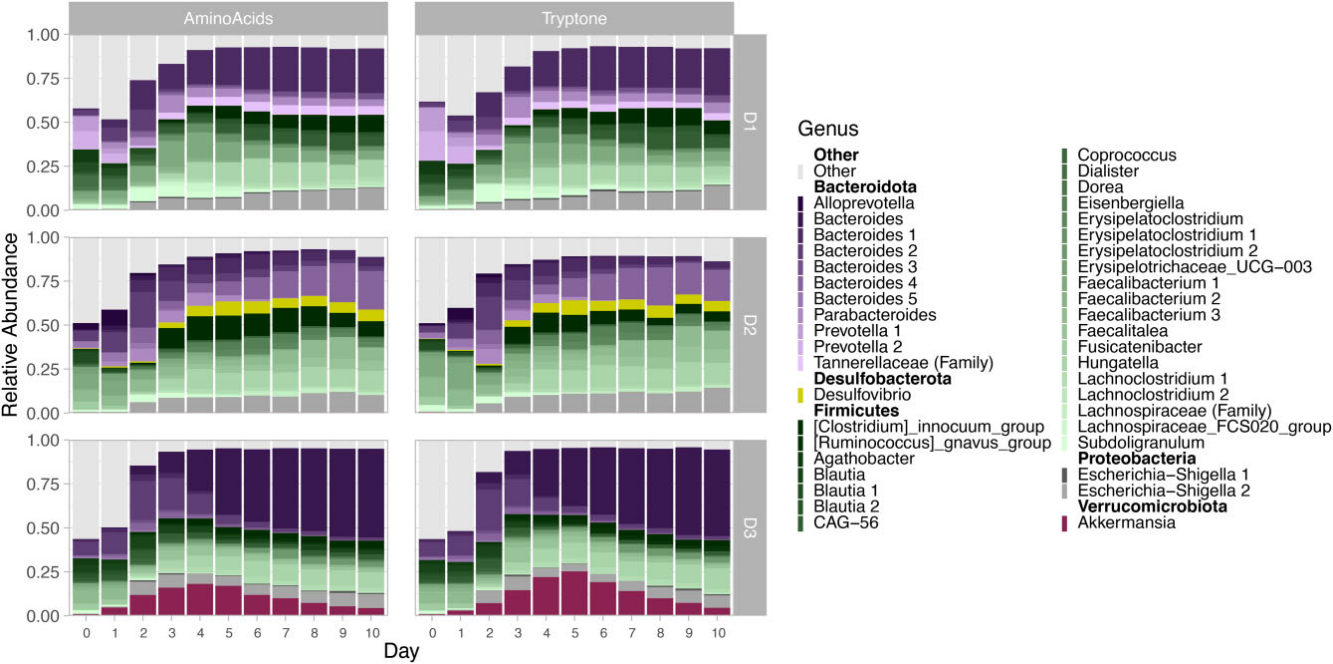
Relative abundance of bacterial taxa for each donor over 10 days with supplementation of either amino acids or tryptone. Averaged abundance of three replicate lineages for each donor indicates mucin sustains diverse fermenting consortia over sequential dilutions that is dependent on the initial community. Day 0 (*n* = 1) is the initial fecal inoculum prior to incubation, whereas each subsequent day reflects the average of three lineages after incubation. Each shade represents a distinct ASV classified down to the level of genus, with the color representing a phylum: Bacteroidota (purple), Desulfobacterota (yellow), Firmicutes (green), Proteobacteria (grey), and Verrucomicrobiota (red). Numbers following genus names represent different ASVs. The top 40 ASVs across all donors (Donor 1, D1; Donor 2, D2; and Donor 3, D3) are shown, where remaining low-abundance ASVs are grouped in Other (light grey).

Dominant phyla across all three communities included Bacteroidetes (Bacteroidota), Firmicutes (Bacillota), and Proteobacteria (Pseudomonadota) (Fig. 5, Supplemental Fig. S5). Although there were no shared ASVs within the Bacteroidetes, by day 10 all three donor communities shared ASVs belonging to Firmicutes and Proteobacteria. At the genus level, these included organisms belonging to the *Clostridium*_innocuum group, *Hungatella, Lachnoclostridium, Faecalibacterium*, and *Escherichia–Shigella*.

In addition to shared features of the three communities, each also had unique members. In donor two communities, a bloom of *Desulfobacterota* appeared at day 3 and remained abundant throughout the experiment. On the other hand, donor three displayed a bloom of *Akkermansia* that had the highest abundances on days 4 and 5, but whose population declined until the termination of the experiment on day 10. To identify less-obvious associations of individual taxa with their donor inoculum, we compared the taxa of the day 10 microbial communities using linear discriminant analysis effect size (LEfSe, Supplemental Fig. S6, Supplemental Table S12). Interestingly, some of the organisms that were shared across the three donors were discriminants for a specific donor due to the differential abundance patterns. At the genus level, in addition to *Desulfovibrio*, other discriminating taxa included *Faecalitalea, Merdibacter, and Erysipelatoclostridium* for the donor two consortium (Supplemental Fig. S6). *Akkermansia* was a discriminant of the donor three community, as were *Ruminococcus* and *Oscillospiraceae*. Although donor one did not have any obvious associations with specific taxa in the visualizations, the FCS020_group and *Howardella* (in the *Lachnospiraceae* family), *Prevotella*, and *Megamonas* were all discriminants of this donor. Interestingly, at the ASV level, each donor had multiple discriminating ASVs belonging to the genus *Bacteroides* and family *Lachnospiraceae* (Supplemental Table S12).

## Discussion

Mucins provide an endogenous nutritional resource in the human gut upon which additional nutrients from the hosts diet are overlaid *in vivo*. As such, mucins are one of the most consistent nutritional resources available to gut microorganisms. Although many individual mucin-degrading bacteria have been identified (Tailford et al. 2015), we wanted to determine the selectivity of mucin across distinct donor communities and begin to understand the fundamental ecology of mucin degradation by gut microbiota. Given that each human hosts a taxonomically distinct gut microbiota, we used fecal samples from three unrelated donors to select for mucin-degrading communities over 10-days of sequential passage and assess community composition and metabolic output using 16S rRNA gene sequencing and SCFA quantification, respectively. Although mucin is a glycoprotein, the ability of an organism or community to access and metabolize the relatively simple protein backbone likely varies. Therefore, to determine whether the provision of amino acids or oligopeptides would differentially impact community structure, replicate communities were developed on either tryptone or a proteinogenic amino acid mixture. At the conclusion of the experiment, we obtained steady-state mucin-degrading communities with distinct compositions across donors and minor compositional differences within a donor cultivated with either amino acid source. While similarities in final community composition did exist, notable taxonomic differences across donors included the presence of the mucin-degrading specialist *Akkermansia* in the donor three community and the presence of the sulfate-reducing *Desulfovibrio* in the donor two community. Metabolic output differences were most apparent for donor two communities, as they produced significantly less acetate and propionate than the other two communities. These findings reinforce the concept that the gut microbiota is taxonomically distinct, yet retains many microbial members that are capable of growth on complex carbohydrates such as mucin. Although all three communities had robust growth on mucin, the taxonomic differences result in distinct metabolic outputs, suggesting that the mechanisms and efficiencies of mucin degradation are important for understanding how this community-level process impacts human health across individuals.

Notable taxonomic similarities across developed communities include the prevalence of *Bacteroides* in all three final communities despite not a single ASV from this genus being shared across donors. Although some members of the *Bacteroides* have known capacity for mucin degradation, such as *B. fragilis* and *B. thetaiotaomicron*, most are not specialized on this carbon source and instead target dietary fibers (Salyers et al. 1977, Pudlo et al. 2015). However, the breadth of GHs harbored by these organisms enables subsistence on mucin in the absence of dietary fibers (Glover et al. 2022, Sonnenburg et al. 2005) or during strong competition by other community members (Sonnenburg et al. 2006). Within the *Bacteroides* populations we note two interesting observations across donors; one, that each donor’s microbiota contained an ASV assigned as *B. vulgatus* that had an initial bloom and then disappeared from the communities; and two, there were different dominant *Bacteroides* ASVs by day 5 that were maintained through the remainder of the experiment. *B. vulgatus* is not a known mucin degrader and likely requires other members of the community to liberate sugars from complex substrates; for example, cocultures with *B. ovatus* on inulin have demonstrated growth dependency of *B. vulgatus* on *B. ovatus*, likely mediated through extracellular GHs (Rakoff-Nahoum et al. 2016). The other *Bacteroides* that dominated donor communities one and two after day 5 could not be classified beyond genus with the SILVA database. However, manually using NCBI BLAST to assign and confirm taxonomy of these sequences (Supplemental Table S11), we found that they closely matched *B. caccae* (community one), *B. thetaiotaomicron* (community two), and confirmed the sequence of *B. fragilis* (community three). All three of these dominant Bacteroides are known to degrade mucin (Desai et al. 2016), suggesting that each donor has a unique *Bacteroides* profile capable of mucin consumption that would enable survival in the absence of dietary fiber. It would be interesting to identify the full spectrum of mucin degrading consortia, specifically with respect to known mucin degraders, within a larger study population or across the same individual to determine whether these microorganisms are retained throughout the life of the host.

After 10 consecutive days of growth on mucin, each donor community maintained ∼30% of its initial diversity, demonstrating that a complex glycoprotein like mucin can sustain relatively diverse microbial communities. While some members of these communities are certainly primary mucin degraders, many of the taxa observed are not. Instead, diversity is at least partially maintained by generalist bacteria feeding on mucin sugars liberated by primary degraders and by bacteria cross-feeding on fermentation end products (Koropatkin et al. 2012). For example, strains of *B. thetaiotaomicron* are able to liberate sialic acid from mucins but unable to import and catabolize it for carbon and energy (Marcobal et al. 2011). Similarly, *B. bifidum* releases mucin monosaccharides, such as fucose, that support other members of the gut microbiota (Bunesova et al. 2018). *Faecalibacterium*, which is a genus of Firmicutes observed in all final donor communities here, is one example of an organism able to use mucin sugars (i.e. GlcNAc) but not intact mucin oligosaccharides (Lopez-Siles et al. 2012). In addition to the liberated monosaccharides, other bacteria could be sustained by fermentation end products. For example, acetate produced by *A. muciniphila* during mucin fermentation can support the growth of the butyrogenic *Eubacterium hallii in vitro* (Belzer et al. 2017). How prevalent this type of cross-feeding across the three donor communities is unknown but could play a significant role in the final amount of SCFAs observed here. Although it is difficult to do more than hypothesize about these metabolic interactions based on 16S rRNA gene sequencing alone, these types of syntrophic interactions are important drivers of overall microbiome function and gut health. Future work should look more directly at these interactions using a multiomics approach of these communities and also coculture experiments.

Beyond sugar liberation, primary mucin degraders could also sustain other functional groups of bacteria, including sulfate reducers, as suggested by a bloom of *Desulfovibrio* sp. in the donor two community. Many mucin oligosaccharides are capped with sulfate groups that must be removed using sulfatase enzymes before accessing the underlying sugar moieties (Katoh et al. 2017, Luis et al. 2021). Although we do not know the functional capabilities of the strain observed here, other *Desulfovibrio* spp. from the human gut do not produce sulfatase enzymes capable of this activity (Rey et al. 2013). This suggests that other members of the community must liberate sulfate to sustain the *Desulfovibrio* bloom observed in the donor 2 community. For example, several species belonging to the Bacteroides are capable of removing sulfate groups from mucin (Luis et al. 2021, Ulmer et al. 2014). We propose that the *Desulfovibrio* in community two reduces liberated sulfate into hydrogen sulfide, as has been previously demonstrated in coculture experiments (Rey et al. 2013). Although we did not measure sulfate accumulation in this experiment, we expect a buildup of sulfate in the donor 1 and 3 communities because of the absence on known sulfate reducers. Where residual sulfate in the colon is likely excreted and is unlikely to affect the host (Florin et al. 1991), high levels of hydrogen sulfide have strong links to colorectal cancer (Wolf et al. 2022). Our study points to the importance of future work aimed at understanding the full metabolic profile of mucin-degrading communities.

Although there were similar trends in community composition across all three donors, of particular interest was the community containing the known mucin-degrading specialist, *Akkermansia. Akkermansia* are widely regarded as beneficial members of the gut microbiota and are known to specialize on mucin glycoproteins because of auxotrophies for GlcNAc and threonine, which are both prevalent in mucin (van der Ark et al. 2018). An initial bloom of *Akkermansia* was observed to represent ∼20% of the community by day 5, which then dwindled by day 10. This community also contained an ASV assigned as *B. fragilis*, another well-established mucin degrader that increased in abundance as *Akkermansia* decreased, suggestive of competition (Roberton and Stanley 1982, Huang et al. 2011). This apparent competition between *Akkermansia* and *B. fragilis* may be strain- or condition-dependent. Recently, the genus *Akkermansia* has been classified into four species-level phylogroups (AmI–AmIV) based on phylogenomic analyses (Guo et al. 2017, Kirmiz et al. 2020). These genotypic differences are recapitulated in phenotypes, for example, where specific phylogroups of *Akkermansia* synthesize vitamin B_12_, potentially outcompeting other organisms in a vitamin B_12_-deficient environment (Kirmiz et al. 2020). Other differences may be directly related to mucin metabolism, where phylogroups AmII, AmIII, and AmIV retain a larger number of GHs related to human milk oligosaccharide metabolism, which has structural similarity with mucin (Luna et al. 2022, Becken et al. 2021). Although the genomic patterns differ across *Akkermansia*, these differences are unidentifiable within the V4 region of the 16S rRNA gene studied here. Furthermore, strain-level diversity analyses within *B. fragilis* have identified repeated mutations across individuals in polysaccharide utilizing loci, suggesting continued within-person evolution, which may be directed by competition with other members of the microbiota, such as other members of the *Bacteroides* or, in the case of mucin, *Akkermansia* (Zhao et al. 2019).

Each donor community was developed in triplicate while maintaining individual lineages with little variability in taxonomy, diversity, and composition across replicates throughout the experiment. We interpret these observations to suggest that mucin as the sole carbon source is a strong selective force driving deterministic community assembly, though the stabilized community selected depended greatly upon initial inoculum. These findings are in line with other sequential cultivation experiments in which diverse and replicable communities have been developed on mucins (Lou et al. 2023) and arabinoxylans (Yao et al. 2023) under high dilution pressure and short incubation times, though unlike mucins, community structures on arabinoxylans converge across donors (Yao et al. 2023). The complexity in glycosidic bonds, which sustain greater species diversity (Midani and David 2023), are likely to drive this selection and limit the ability for any one organism to dominate. Although the dilution rate and sampling times may select for fast growing organisms (Adamberg and Adamberg 2018), these same organisms are expected to thrive in native conditions, under which the average gut transit time is 24 h (Asnicar et al. 2021, Nandhra et al. 2020). In our experiments, the mucin solution is first dialyzed to remove small oligosaccharides (<12 kDa) and then filtered (<0.2 uM), resulting in large, soluble glycoproteins that are likely processed extracellularly before transport. This mucin preprocessing step is likely effective, as simple sugars, when present in these types of cultivation experiments, can result in communities that are dominated by one or two species (Lou et al. 2023, Yao et al. 2020, Wu et al. 2023). While the composition of the porcine gastric mucin used in these experiments is unknown, a previous study found similarities and differences in both protein and carbohydrate composition compared to human mucins (Miner-Williams et al. 2009). Porcine gastric mucin tends to have a higher percentage of carbohydrates by weight while human mucins are more protein rich. In terms of which sugars are present, porcine gastric mucin tends to have more GlcNAc, galactose, and fucose as compared to human colonic mucins. Despite these differences, others have noted that mucin and mucin glycans originating from both porcine and human sources result in the ability to support similar diverse communities (Wu et al. 2023). Additionally, similar filtration and dialysis steps on labeled porcine mucin result in large glycoproteins that are metabolized by mucin degrading specialists (Davey et al. 2023). Therefore, it will be interesting to determine the inflection point of mucin complexity at which the transition from single-organism culture to multispecies consortium occurs and if mucin source influences community development.

In addition to nutrient availability, the absence of specific nutrients in these systems may be influential on selection and community assembly. Auxotrophies, both for vitamins and amino acids, are highly prevalent across many microbial communities (Yu et al. 2022). In similar sequential batch-culture cultivation experiments, the relief of these auxotrophies has a demonstrable effect on final community composition (Yao et al. 2020). In the experiments here, common vitamin auxotrophies were alleviated through addition of the ATCC vitamin supplement. However, in hand with amino acid auxotrophies, the abilities of an organism to scavenge amino acids into its cells vary; further, some organisms produce exopeptidases capable of breaking down complex proteins into smaller fragments, as dipeptides or free amino acids, which are then transported into the cell (Zhang et al. 2022). Although the mucin backbone does provide a potential source of amino acids, not all bacteria produce peptidases able to cleave the mucin backbone. Therefore, to ameliorate any amino acid auxotrophy without providing additional carbon sources, we provided a low concentration of free amino acids or tryptone in the fermentation medium and assessed whether the complexity of amino acids influenced the final microbial community structure. The lack of ASVs discriminated by LEfSe analysis comparing the final communities cultivated with the two amino acid sources is in line with observations of bacterial growth in minimal amino acid-free media (Price et al. 2018) and cross-feeding interactions in complex systems (Du et al. 2022). These dynamic interactions by other members of the microbial community may be species- or strain-specific (Ashniev et al. 2022), which argues for a comprehensive analysis of the genomic capacity of these organisms using metagenomics and assembly of species’ genomes to fully determine whether organisms with auxotrophies were maintained in these experiments.

### In summary

Identification of individual mucin-degrading consortia necessitated the use of *in vitro* models, in which mucin was the sole carbon source. For example, in *in vivo* studies in both animals and humans, the nutritional role of mucins is obscured by dietary conditions. Therefore, the batch-model system used in this research provides essential advantages to identify the ecological networks of mucin degradation. However, variability in mucin structure and composition along the length of the intestinal tract (Robbe et al. 2004), secretion of antimicrobial peptides (Bevins 2007), and spatial organization of individual microbes (Mark Welch et al. 2017) are remaining aspects of mucin degradation that are host-relevant and difficult to replicate *in vitro*. Development of these communities *in vitro* using a top-down approach allows the examination of microbe–microbe interactions while accounting for naturally occurring strain heterogeneity amongst core bacterial groups, an advantage not afforded by bottom-up approaches that rely on assembling communities using type strains originating from different sources.

## Supplementary Material

fiae078_Supplemental_Files

## Data Availability

All raw sequencing data in FASTQ format are available in the NCBI Sequence Read Archive (SRA) BioProject database under accession number PRJNA956585 as BioSamples SAMN34224450–SAMN34224644
