# Supplementary material for: Enrichment and characterization of human-associated mucin-degrading microbial consortia by sequential passage
Source: FEMS Microbiol Ecol. 2024 May 24;100(7):fiae078. doi: 10.1093/femsec/fiae078 (PMC11180985; doi:10.1093/femsec/fiae078)
Supplement: fiae078_Supplemental_Files [file fiae078_supplemental_files.zip › Mucin Comm Supp data Figure Legends v3.docx]

Supplemental Figure Legends

Supplemental Figure 1. Final (day 10) optical density and pH measurements of mucin degrading consortia supplemented with different amino acid sources. With the exception of Donor 2 acid production, the optical density (OD, A) and acid production (pH, B) of mucin degrading consortia from the same donor grown with different amino acid sources were consistent. Averages of three lineages for each final donor community (Donor 1, D1; Donor 2, D2; Donor 3, D3) are displayed with error bars representing standard deviation as calculated in R. Statistically significant differences are calculated by Tukey’s multiple comparisons test with P<0.05. Symbol style: nonsignificant (ns), 0.05 (*), 0.01 (**), 0.001(***) and <0.0001(****)

Supplemental Figure 2. Short chain and branched chain fatty acid profiles of mucin degrading consortia after 10 days of sequential transfer. Mucin degrading consortia from donors one and three produced the higher concentration of isobutyrate (A) and isovalerate (B) than the donor two consortium, irrespective of amino acid source. Mean, first and third quartiles for each final donor community (Donor 1, D1; Donor 2, D2; Donor 3, D3) are represented. Statistically significant differences are calculated by Tukey’s multiple comparisons test. Symbol style: nonsignificant (ns), 0.05 (*), 0.01 (**), 0.001(***) and <0.0001(****)

Supplemental Figure 3. Bray Curtis dissimilarity calculated between consecutive days for each lineage. Community structure plateaued between days five and seven, independent of amino acid source. Smoothed conditional means calculated for cultures grown with amino acids (grey) or tryptone (orange).

Supplemental Figure 4. Percent loss of ASVs as compared to the number of ASVs in the fecal inoculum (day zero) when supplemented with amino acids or tryptone across 10 days. Percent loss was approximately equal across donor samples, irrespective of amino acid source. The mean, first and third quartiles for each donor (Donor 1, D1; Donor 2, D2; Donor 3, D3) for

amino acids provided as an equimolar mix (amino acids, blue) or peptides (tryptone, pink) are shown.

Supplemental Figure 5. Relative abundance of bacterial taxa for each donor over 10 days with supplementation of either amino acids or tryptone. Averaged abundance of three replicate lineages for each donor indicates mucin sustains diverse fermenting consortia over sequential dilutions that is dependent on the initial community. Day zero (n=1) is the initial fecal inoculum prior to incubation, whereas each subsequent day reflects the average of three lineages after incubation. Each shade represents a distinct ASV classified down to the level of species, with the dominant color representing a Family, separated by Bacillota (A), Bacteroidota (B), Desulfobacterota (C), Proteobacteria (D), and Verrucomicrobiota (E). Numbers following species names represent different ASVs. The top fifty ASVs across all donors (Donor 1, D1; Donor 2, D2; Donor 3, D3) are shown.

Supplemental Figure 6. Linear discriminant analysis differentiating the combined day 10 communities from both amino acid sources across the donors indicate that final consortia had multiple members unique to each donor. Taxa with Linear Discriminant Analysis (LDA) scores >3.0 are shown at the level of genera.

Supplemental Table 1. Composition of tryptone and the prepared amino acid mix is different between supplements. The final concentration of amino acids in the tryptone-supplemented cultures is calculated based on published estimated percentages of each amino acid [(tryptone CAS 91079-40-2)].

Supplemental Table 2. Beta diversity between selected samples across two sequencing runs and between the final consortium across three donors was compared by ADONIS (PERMANOVA). Microbial community composition was not affected by sequencing run but was different across three donors after 10 days of sequential passage. Abbreviations: df, degrees of freedom; SS, sum of squares. Symbol style: nonsignificant (ns) and 0.001 (**).

Supplemental Table 3. Beta diversity across all donors and within a donor across amino acid source and days was compared by PERMANOVA to determine significant associations. Abbreviations: DF, degrees of freedom; SumOfSqs, sum of squares; R2, partial R squared; F, pseudo-F statistic; Pr(>F), P-value for F statistic.

Supplemental Table 4. Community dissimilarity (Bray Curtis) was compared across each day of development using pairwise PERMANOVA to determine when dissimilarity plateaued for each donor with combined amino acid conditions. Highlighted rows indicate tests between consecutive days and a non-significant (ns) result was used to determine when stability was achieved.

Supplemental Table 5. Alpha diversity between amino acid source and days across three donors was compared by independent-measures ANOVA to determine significant associations. Abbreviations: DFn and Dfd, degrees of freedom in the numerator and denominator, respectively; F, F-calue; p, p-value; ges, Generalized Eta-Squared measure of effect size.

Supplemental Table 6. Community richness (ASV counts) was compared across each day of development using Tukey’s multiple comparisons test to determine when diversity plateaued for each donor under both media conditions. Highlighted rows indicate tests between consecutive days and a non-significant (ns) result was used to determine when stability was achieved.

Supplemental Table 7. Community diversity (Shannon entropy) was compared across each day of development using Tukey’s multiple comparisons test to determine when diversity plateaued for each donor under both media conditions. Highlighted rows indicate tests between consecutive days and a non-significant (ns) result was used to determine when stability was achieved.

Supplemental Table 8. Community richness (ASV counts) was compared within each donor across amino acid source during each day of development using Tukey’s multiple comparisons test.

Supplemental Table 9. Community diversity (Shannon entropy) was compared within each donor across amino acid source during each day of development using Tukey’s multiple comparisons test.

Supplemental Table 10. Percent of ASVs lost relative to day 0 was compared across each day of development using Tukey’s multiple comparisons test to determine when diversity plateaued for each donor under both media conditions. Highlighted rows indicate tests between consecutive days and a non-significant (ns) result was used to determine when stability was achieved.

Supplemental Table 11. NCBI BLAST confirmed and assigned taxonomy of sequences belonging to the phylum Bacteroidota. Top hit for NCBI classification was selected and compared to the Qiime annotation. Dominant ASVs from each community are highlighted in bold with an asterisk adjacent to the NCBI match.

Supplemental Table 12. Linear discriminant analysis differentiating the combined day 10 communities from both amino acid sources across the donors indicate that all final consortia had ASVs belonging to Bacteroidaceae, Lachnospiraceae, and Oscillospiraceae. Taxa with Linear Discriminant Analysis (LDA) scores >3.0 are shown at the level of ASV.
