# Supplementary material for: Enrichment and characterization of human-associated mucin-degrading microbial consortia by sequential passage
Source: FEMS Microbiol Ecol. 2024 May 24;100(7):fiae078. doi: 10.1093/femsec/fiae078 (PMC11180985; doi:10.1093/femsec/fiae078)
Supplement: fiae078_Supplemental_Files [file fiae078_supplemental_files.zip › Supp data Figure4.pdf]

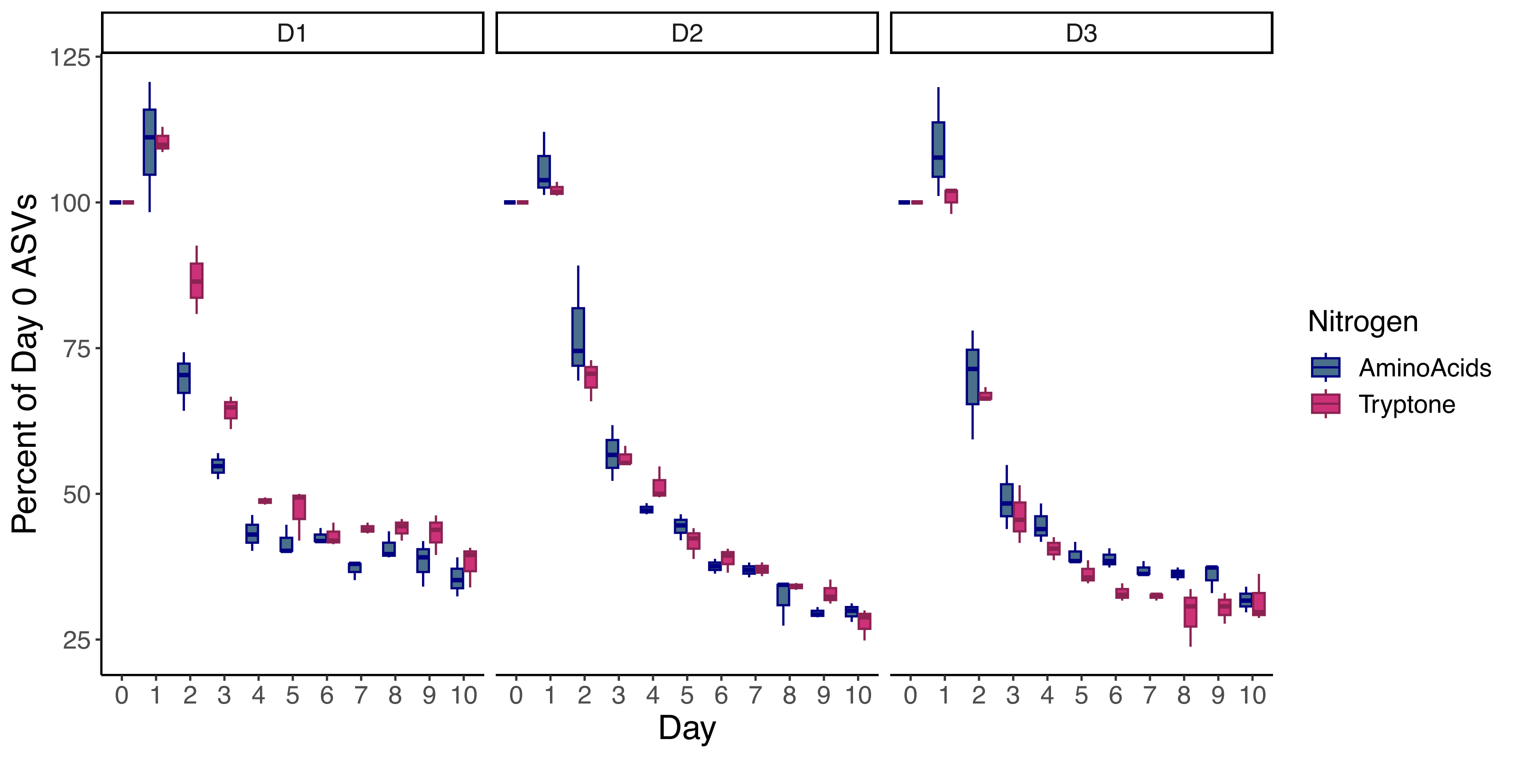
D1
D2
D3
125
100
75
50
25
Percent of Day 0 ASVs
0
1
2
3
4
5
6
7
8
9
10
Day
Nitrogen
AminoAcids
Tryptone
