# Supplementary material for: Enrichment and characterization of human-associated mucin-degrading microbial consortia by sequential passage
Source: FEMS Microbiol Ecol. 2024 May 24;100(7):fiae078. doi: 10.1093/femsec/fiae078 (PMC11180985; doi:10.1093/femsec/fiae078)
Supplement: fiae078_Supplemental_Files [file fiae078_supplemental_files.zip › Supp data Table1.pdf]

Amino Acid	mw	Code	Tryptone (estimated) Percent	uM	Amino Acid Mix uM
L-Alanine	89.09	Ala	2.8	31.43	10
L-Arginine	174.2	Arg	3.2	18.37	10
L-Asparagine	132.1	Asp	6.7	50.53	10
L-Aspartic Acid	133.1	Asx			10
L-Cysteine	121.2	Cys	0.3	2.48	10
L-Glutamic Acid	147.1	Glu	18	122.78	10
L-Glutamine	146.1	Glx			10
Glycine	75.07	Gly	1.8	23.98	10
L-Histidine	155.2	His	2.4	15.46	10
L-Isoleucine	131.2	Ile	4.7	35.82	10
L-Leucine	131.2	Leu	7.7	58.69	10
L-Lysine Monohydrochloride	182.7	Lys	7	38.31	10
L-Methionine	149.2	Met	2.5	16.76	10
L-Phenylalanine	165.2	Phe	4.1	24.82	10
L-Proline	115.1	Pro	9.1	79.06	10
L-Serine	105.1	Ser	5.1	48.53	10
L-Threonine	119.1	Thr	3.9	32.75	10
DL-Tryptophan	204.2	Trp	1	4.90	10
L-Tyrosine	181.2	Tyr	1.3	7.17	10
L-Valine	117.2	Val	6	51.19	10
