# Supplementary material for: Enrichment and characterization of human-associated mucin-degrading microbial consortia by sequential passage
Source: FEMS Microbiol Ecol. 2024 May 24;100(7):fiae078. doi: 10.1093/femsec/fiae078 (PMC11180985; doi:10.1093/femsec/fiae078)
Supplement: fiae078_Supplemental_Files [file fiae078_supplemental_files.zip › Supp data Table11.pdf]

| Dominant ASV | ASV | NCBI Match | Qiime annotation | | |
|---|---|---|---|---|---|
| | | | Family | Genus | Species |
| | 0cc2f79a3e3d31e1d291a8e083e611ab | Bacteroides_finegoldii | Bacteroidaceae | Bacteroides | |
| | 1b6ffc0a05a7be8d4f4d95425832d107 | Bacteroides_caccae | Bacteroidaceae | Bacteroides | Bacteroides caccae |
| | 1c3b3d6b5540ae4459f826420b48da10 | Parabacteroides_merdae | Tannerellaceae | Parabacteroides | Parabacteroides merdae |
| | 1ef97ff90aed6940d9fbd604ce20da5d | Bacteroides_caccae | Bacteroidaceae | Bacteroides | Bacteroides caccae |
| | 20cf13f228f8d16b499e9493dd2ee208 | Bacteroides_uniformis | Bacteroidaceae | Bacteroides | |
| | 2d3f536ce7257608ddf6f44a800c218b | Bacteroides_nordii | Bacteroidaceae | Bacteroides | |
| | 2ed7801e4cef3b35f4140acd2f1101e7 | Bacteroides_intestinalis | Bacteroidaceae | Bacteroides | Bacteroides intestinalis |
| | 3a5dc0b707e689a2beeeb1e6c17d9f6c | Bacteroides_clarus | Bacteroidaceae | Bacteroides | Bacteroides clarus |
| | 3cf6dd6edede75f439d74272791b67ac | Parabacteroides_pekinense | Tannerellaceae | Parabacteroides | |
| | 4f13b31757426267a1b923846bcfe43d | Bacteroides_uniformis | Bacteroidaceae | Bacteroides | Bacteroides uniformis |
| | 507d64a7f56fccec9f413747b5842972 | Parabacteroides_distasonis | Tannerellaceae | Parabacteroides | |
| | 50da935cb4343ba6c21695a03d5353ca | Bacteroides_stercoris | Bacteroidaceae | Bacteroides | Bacteroides stercoris |
| | 5401edfc1902e0f71700034ae1f78eb1 | Bacteroides_ovatus | Bacteroidaceae | Bacteroides | |
| | 6501054fb595c538eb4033530612d4a3 | Bacteroides_stercoris | Bacteroidaceae | Bacteroides | Bacteroides stercoris |
| | 6521f34e85eb9034e3bd9d4e58dff50a | Bacteroides_ovatus ; Bacteroides_xylanisolvens | Bacteroidaceae | Bacteroides | |
| | 6bd982a66b0c74116b5c1cd9b824c113 | Bacteroides_cellulosyliticus | Bacteroidaceae | Bacteroides | |
| | 6e14788f3f3cb78ee7fa0348a0c2cf46 | Bacteroides_plebeius | Bacteroidaceae | Bacteroides | Bacteroides plebeius |
| | 7b7f3706e2803ced3a3eddfa7887e7dc | Bacteroides_sartorii | Bacteroidaceae | Bacteroides | Bacteroides sartorii |
| | 84618cc630790f97e68fed6ac3a55c68 | Bacteroides_coprocola | Bacteroidaceae | Bacteroides | Bacteroides coprocola |
| | 8974faa17be540e9a3a18be3919846bb | Bacteroides_thetaiotaomicron | Bacteroidaceae | Bacteroides | |
| **D1** | **8f2ddb8d1729fc35598d70fbcbd1b1af** | **Bacteroides_thetaiotaomicron*** | **Bacteroidaceae** | **Bacteroides** | |
| | 97d34dbaf75b1ffbb56b6d7b8db933bf | Bacteroides_ovatus | Bacteroidaceae | Bacteroides | |
| | b0c83858accb36fae1309230e71f31a3 | Bacteroides_coprocola | Bacteroidaceae | Bacteroides | Bacteroides coprocola |
| | b57b0a37bd78a259c27a9cc823483311 | Bacteroides_vulgatus | Bacteroidaceae | Bacteroides | Bacteroides vulgatus |
| | b5bf467bb53eb088c18e684d8656f904 | Bacteroides_clarus | Bacteroidaceae | Bacteroides | Bacteroides clarus |
| | bd125abcbb6d6ed03a18c0515fee1db4 | Parabacteroides_distasonis | Tannerellaceae | Parabacteroides | |
| | c06b712348f8542720421722120be38e | Bacteroides_uniformis | Bacteroidaceae | Bacteroides | |
| | ca1392eaa969b2980a69cc34a327e112 | Bacteroides_dorei | Bacteroidaceae | Bacteroides | Bacteroides vulgatus |
| | cece12865e81c96a523b3bb6a4511561 | Bacteroides_vulgatus | Bacteroidaceae | Bacteroides | Bacteroides vulgatus |
| **D2** | **d051e46a2ac73aead2b4a876701c9862** | **Bacteroides_caccae*** | **Bacteroidaceae** | **Bacteroides** | |
| | d0a48a8fafdce31a7583434c537587c2 | Parabacteroides_distasonis | Tannerellaceae | Parabacteroides | |
| **D3** | **d8999f85296a9d9f0eb3a48834df9d46** | **Bacteroides_fragilis*** | **Bacteroidaceae** | **Bacteroides** | **Bacteroides fragilis** |
| | db79739512d1a8e2ea6457e99ff96b8f | Parabacteroides_distasonis | Tannerellaceae | Parabacteroides | Parabacteroides distasonis |
| | e1dd787a45b6582d7dc397295640fed0 | Bacteroides_clarus | Bacteroidaceae | Bacteroides | Bacteroides clarus |
| | f00b40d9dfd549fc8240a0dec837bf53 | Bacteroides_sartorii | Bacteroidaceae | Bacteroides | Bacteroides sartorii |
