# Supplementary material for: Enrichment and characterization of human-associated mucin-degrading microbial consortia by sequential passage
Source: FEMS Microbiol Ecol. 2024 May 24;100(7):fiae078. doi: 10.1093/femsec/fiae078 (PMC11180985; doi:10.1093/femsec/fiae078)
Supplement: fiae078_Supplemental_Files [file fiae078_supplemental_files.zip › Supp data Table12.pdf]

Donor 1

Family	Genus	ASV	LDA	pval
Bacteroidaceae	Bacteroides	d051e46a2ac73aead2b4a876701c9862	5.09841134	0.00459133
Lachnospiraceae	Hungatella	35da7d6c16006d7f9b1c9ef4554163ff	4.45224349	0.01060526
Erysipelotrichaceae	Clostridium	718be274e36083f02047feb0afbd6207	4.45068883	0.00257957
Enterobacteriaceae	Escherichia-Shigella	c3342d2a9a35fb23f494d299efedfcb9	4.41040705	0.03405636
Lachnospiraceae	CAG56	dc022be46eee04d339c608d9c68b02bd	4.35902094	0.00938592
Tannerellaceae		de17fa4e217c05126bad3d8e4a5ed1ae	4.35191544	0.00222836
Tannerellaceae	Parabacteroides	3cf6dd6edede75f439d74272791b67ac	4.27862824	0.00657416
Ruminococcaceae	Faecalibacterium	d7b8a25da82bccc48f91f25966c10940	4.2624306	0.02520462
Bacteroidaceae	Bacteroides	97d34dbaf75b1ffbb56b6d7b8db933bf	4.20561143	0.00644706
Lachnospiraceae	Lachnoclostridium	d5b22a367668019c66d346e758f7a1ee	4.01602903	0.00965896
Erysipelatoclostridiaceae	Erysipelatoclostridium	484951ad2d9d3b32e47b91ac09845cad	3.97597505	0.00222836
Lachnospiraceae	Blautia	cd2d1c880543440df16252c701b79fdb	3.95687298	0.01005916
Lachnospiraceae	Lachnoclostridium	8f62d545ecb1feb5e80c53246d162aff	3.7567431	0.0419789
Lachnospiraceae	Clostridium	68d64bdc69fca911af1825a7299db9d1	3.69693082	0.00222836
Selenomonadaceae	Megamonas	43303b616239cfb4ac93969fa8511f38	3.65384988	0.00098175
Enterobacteriaceae	Escherichia-Shigella	7bcfb947c7e3082a424b0b844df33b48	3.4766273	0.01879445
Oscillospiraceae	UCG3	95501a89a3915469692bdb7941c11379	3.4622064	0.02867516
Lachnospiraceae	GCA900066575	0b96cceb5603453ce713f98fbced89b8	3.4489434	0.01599311
Tannerellaceae	Parabacteroides	1c3b3d6b5540ae4459f826420b48da10	3.44756817	0.0068668
Acidaminococcaceae	Phascolarctobacterium	26d5c4614643a0b9609257f324bbcaae	3.37534951	0.00314647
Lachnospiraceae	Lachnoclostridium	f3ddb1bdc3ca0c36f0543346f367de3b	3.30705088	0.00309802
Lachnospiraceae	Eubacterium	7ab180612716a7fa058f4b230bfdf8b4	3.29780868	0.00679977
Ruminococcaceae	Faecalibacterium	2358df28ab58699505715869111dddbd	3.28748627	0.00284859
Acidaminococcaceae	Phascolarctobacterium	25b18ecaa3130943bc641a91603166b0	3.28113066	0.00325952
Ruminococcaceae	Subdoligranulum	24bdbf1a7778b804aadd472704d1cbbd	3.26780492	0.00325952
Lachnospiraceae		4339ba364f4fea794ea315585b9f181f	3.26684442	0.0046749
Sutterellaceae	Sutterella	6e4236a1abc321b6fe8131a9b686f8d9	3.20373279	0.00098175
Lachnospiraceae	Lachnoclostridium	8b13d348319c4dbde681073f39027d43	3.19681786	0.0046749
Oscillospiraceae	UCG003	4a31d423711776c23a965d7b276222a2	3.18679873	0.01234337
Ruminococcaceae	Subdoligranulum	7b0ae3dee754648b1de84fb4bf1df54a	3.17560256	0.01709964
Bacteroidaceae	Bacteroides	2d3f536ce7257608ddf6f44a800c218b	3.1619665	0.00606684
Ruminococcaceae	Subdoligranulum	2d84b211b3d07ca6280c3e0ff64b0705	3.15791587	0.00325952
Lachnospiraceae		b1d59ccbfd08400f257921ca431858c3	3.12938695	0.00896207
Erysipelatoclostridiaceae	Erysipelotrichaceae	500d57e4d12cc186a53f984c1496f257	3.12479957	0.01709964
Lachnospiraceae	Lachnoclostridium (Clostridium)	a18434a7badd84195a6bd5a5e88a2a74	3.08389997	0.00045368
Oscillospiraceae		716f58b747dbbc494e82ea87d24c7346	3.01032963	0.00325952
Lachnospiraceae	Lachnoclostridium (Clostridium)	7fb9ec6296b13a71a6341ef0be801f54	3.00077864	0.00325952

Donor 2

Family	Genus	ASV	LDA	pval
Bacteroidaceae	Bacteroides	8f2ddb8d1729fc35598d70fbcbd1b1af	4.93678717	0.00511431
Lachnospiraceae	Hungatella	ba56a5b65120693452a96ae6af6cf0b7	4.66056642	0.00648127
Desulfovibrionaceae	Desulfovibrio	9767e7ed0d987589b2863b69a8808082	4.46154452	0.01708197
Erysipelotrichaceae	Faecalitalea	ee983f5bc9efbee9900a2a6e65db0bdd	4.39790282	0.02752003
Enterobacteriaceae		325894a0b9a19f4bd2d045ecd2444d80	4.30031115	0.00601142
Bacteroidaceae	Bacteroides	50da935cb4343ba6c21695a03d5353ca	4.28154017	0.01172921
Erysipelatoclostridiaceae	Erysipelatoclostridium (Clostridium)	dbadd8935a42590900724f074cf3dabd	4.07507057	0.02104068
Bacteroidaceae	Bacteroides	6501054fb595c538eb4033530612d4a3	4.00335955	0.01165638
Lachnospiraceae		0599be2ab8aa7822fcafaa68f1ac421e	3.81257508	0.01165638
Lachnospiraceae	Eisenbergiella	5ed4d2bf393c9df61b330562da657549	3.75660105	0.01781934
Desulfovibrionaceae	Desulfovibrio	db38e88f9de7e01d29ee1d5baa188eca	3.75538244	0.00601142
Oscillospiraceae	Intestinimonas	6ac36eccac4f7fdc9aba11dccb40a4ac	3.35452255	0.00844611
Lachnospiraceae	Lachnoclostridium	4cac504948b474e0f0c87e95334b5200	3.30614732	0.00601142
Lachnospiraceae		1affcd51533d87bef4e84e3f6fcf03da	3.25976387	0.01580149
Lachnospiraceae	Ruminococcus	b37c72712ed67b5df6653cd80fae6703	3.22900333	0.00601142
Erysipelatoclostridiaceae	Candidatus Stoquefichus	3d63e31da697f55580940c9e6722b5d6	3.14850935	0.01580149
Erysipelotrichaceae	Merdibacter	9a280626ca1f7a51676796ef67fedd21	3.10241357	0.02104068

Donor 3

Family	Genus	ASV	LDA	pval
Bacteroidaceae	Bacteroides	d8999f85296a9d9f0eb3a48834df9d46	5.32138059	0.00279338
Akkermansiaceae	Akkermansia	438fb020cf251f2c2f4e89552e4a78ff	4.23038384	0.01772391
Erysipelatoclostridiaceae	Erysipelatoclostridium	55275c7900d36914bcebfaf9cdd89063	4.08917542	0.00279338
Lachnospiraceae	Ruminococcus	cd3e52559a9e53dce7721688de8fd9b5	3.89387601	0.00279338
Erysipelotrichaceae	Faecalitalea	26bc9249712e574776fdfbc535e14587	3.8351257	0.00279338
Lachnospiraceae	Blautia	312015c00f6652c04c215fbc47488649	3.79521407	0.04647133
Bacteroidaceae	Bacteroides	5401edfc1902e0f71700034ae1f78eb1	3.79260356	0.00279338
Lachnospiraceae	Eisenbergiella	1893fc5edfb6cf17665c186c5315ec9e	3.79125153	0.00253438
Lachnospiraceae	Blautia	9ca9e80bb08ba22912ea23d7c2ba17fd	3.77273954	0.00098764
Lachnospiraceae	Ruminococcus	385e25b78d2425e47fba70ec065ebc32	3.57163973	0.00279338
Lachnospiraceae		b813cdcad2df04855bbeb3d29247defd	3.49559842	0.00661074
Oscillospiraceae	Flavonifractor	d54e84e50addcb527e4290d59525524f	3.48858348	0.00965265
Lachnospiraceae		5eccc6f6f4334997aa81eed6e4860491	3.38974695	0.00355786
Ruminococcaceae	Incertae Sedis	8b70ae7ae06e4325a443d32067235627	3.22367239	0.04468876
Oscillospiraceae	Intestinimonas	5bb18e57884b5a2f610cea26d0610331	3.19528337	0.01211902
Veillonellaceae	Dialister	51a8d5697d6a6fc543f2562aacc07f79	3.19125307	0.00279338
Streptococcaceae	Streptococcus	abecbcf11dfc066685a87b6428a58a17	3.13527825	0.01211902
Lachnospiraceae	Eubacterium	2e63e8e3f9f0f102f570e736e4b27c69	3.03703199	0.03635662
