# Supplementary material for: Enrichment and characterization of human-associated mucin-degrading microbial consortia by sequential passage
Source: FEMS Microbiol Ecol. 2024 May 24;100(7):fiae078. doi: 10.1093/femsec/fiae078 (PMC11180985; doi:10.1093/femsec/fiae078)
Supplement: fiae078_Supplemental_Files [file fiae078_supplemental_files.zip › Supp data Table2.pdf]

		df	***SS***	***R2***	***F***	***Pr(>F)***	**Perms**
Samples sequenced on run 1 and run 2 (n = 6 per group)	Bray Curtis	*1*	*0.010414*	*0.0032369*	*0.0325*	*0.96 (ns)*	999
	Unweighted	1	0.083236	0.052774	0.5571	0.633 (ns)	999
	Weighted	1	0.0008892	0.0025508	0.0256	0.947 (ns)	999
	Jaccard	1	0.15304	0.044219	0.4627	0.848 (ns)	999
Donor Communities at Day 10 (n = 6 per group)	Bray Curtis	3	3.9001379	0.8928368	44.43502	0.001***	999
	Unweighted	3	1.213736	0.7837684	19.33158	0.001***	999
	Weighted	3	0.39984043	0.8531772	30.99163	0.001***	999
	Jaccard	3	3.9475482	0.8191273	24.15334	0.001***	999
