# Supplementary material for: Enrichment and characterization of human-associated mucin-degrading microbial consortia by sequential passage
Source: FEMS Microbiol Ecol. 2024 May 24;100(7):fiae078. doi: 10.1093/femsec/fiae078 (PMC11180985; doi:10.1093/femsec/fiae078)
Supplement: fiae078_Supplemental_Files [file fiae078_supplemental_files.zip › Supp data Table3.pdf]

		Df	SumOfSqs	R2	F	Pr(>F)
Across All Donors	adonis2(formula = data.dist.BC~Donor, data = Analysis2, permutations = 999)					
	Donor	2	24.051	0.53995	103.87	0.001
	Residual	177	20.492	0.46005		
	Total	179	44.543	1		
Donor 1	adonis2(formula = data.dist.BCD1~Day*Nitrogen, data=D1, permutations = 9999)					
	Day	1	3.2713	0.51917	63.6174	0.0001
	Nitrogen	1	0.1071	0.017	2.0833	0.1154
	Day:Nitrogen	1	0.0429	0.00682	0.8351	0.4189
	Residual	56	2.8796	0.45701		
	Total	59	6.3009	1		
	Donor 2					
Donor 2	adonis2(formula = data.dist.BCD2~Day*Nitrogen, data=D2, permutations = 9999)					
	Day	1	3.8092	0.52145	65.1155	0.0001
	Nitrogen	1	0.1696	0.02321	2.8988	0.0529
	Day:Nitrogen	1	0.0502	0.00688	0.8586	0.4057
	Residual	56	3.276	0.44846		
	Total	59	7.305	1		
Donor 3	adonis2(formula = data.dist.BCD3~Day*Nitrogen, data=D3, permutations = 9999)					
	Day	1	3.9729	0.57695	79.1406	0.0001
	Nitrogen	1	0.0696	0.01011	1.3865	0.2299
	Day:Nitrogen	1	0.0323	0.00469	0.6434	0.5189
	Residual	56	2.8112	0.40825		
	Total	59	6.886	1		
