# Supplementary material for: Enrichment and characterization of human-associated mucin-degrading microbial consortia by sequential passage
Source: FEMS Microbiol Ecol. 2024 May 24;100(7):fiae078. doi: 10.1093/femsec/fiae078 (PMC11180985; doi:10.1093/femsec/fiae078)
Supplement: fiae078_Supplemental_Files [file fiae078_supplemental_files.zip › Supp data Table5.pdf]

	Donor	Effect	DFn	DFd	F	p	p<.05	ges
Observed Features	D1	Day	9	40	200.176	2.26E-30	*	0.978
		Nitrogen	1	40	0.023	0.881		0.000569
		Day:Nitrogen	9	40	2.464	0.024	*	0.357
	D2	Day	9	40	277.916	3.68E-33	*	0.984
		Nitrogen	1	40	13.132	0.00081	*	0.247
		Day:Nitrogen	9	40	1.082	0.397		0.196
	D3	Day	9	40	212.838	6.83E-31	*	0.98
		Nitrogen	1	40	0.005	0.945		0.000119
		Day:Nitrogen	9	40	0.631	0.764		0.124
Shannon Entropy	D1	Day	9	40	366.048	1.63E-35	*	0.988
		Nitrogen	1	40	14.168	0.000537	*	0.262
		Day:Nitrogen	9	40	3.143	0.006	*	0.414
	D2	Day	9	40	108.033	3.35E-25	*	0.96
		Nitrogen	1	40	3.229	0.08		0.075
		Day:Nitrogen	9	40	0.523	0.849		0.105
	D3	Day	9	40	381.887	7.06E-36	*	0.988
		Nitrogen	1	40	1.524	0.224		0.037
		Day:Nitrogen	9	40	1.056	0.415		0.192
Percent of Initial	D1	Day	9	40	207.96	1.07E-30	*	0.979
		Nitrogen	1	40	29.797	2.72E-06	*	0.427
		Day:Nitrogen	9	40	2.34	0.032	*	0.345
	D2	Day	9	40	275.532	4.36E-33	*	0.984
		Nitrogen	1	40	0.41	0.525		0.01
		Day:Nitrogen	9	40	1.611	0.145		0.266
	D3	Day	9	40	193.075	4.57E-30	*	0.977
		Nitrogen	1	40	18.003	0.000127	*	0.31
		Day:Nitrogen	9	40	0.534	0.841		0.107
