# Supplementary figures and images for: Enrichment and characterization of human-associated mucin-degrading microbial consortia by sequential passage
Source: FEMS Microbiol Ecol. 2024 May 24;100(7):fiae078. doi: 10.1093/femsec/fiae078 (PMC11180985; doi:10.1093/femsec/fiae078)

OD

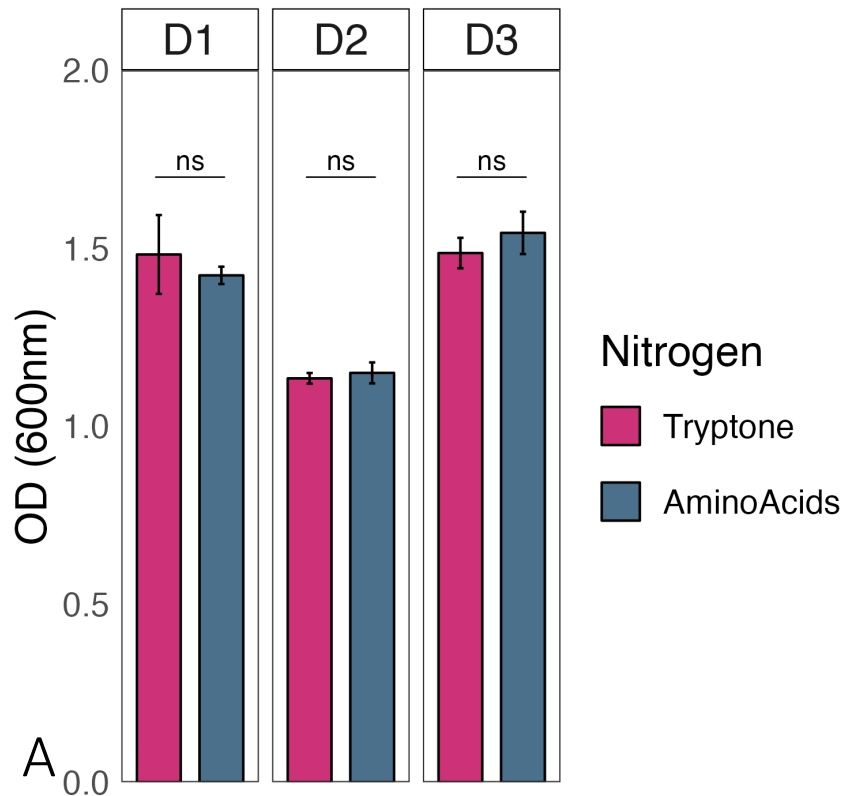

change in pH

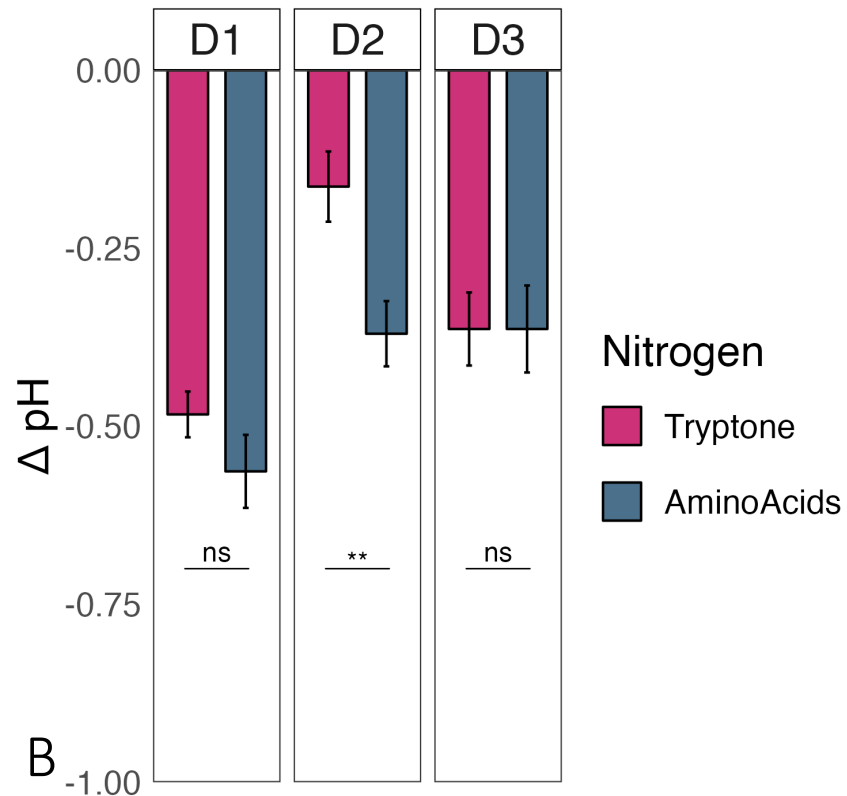

Supplement: fiae078_Supplemental_Files [file fiae078_supplemental_files.zip › Supp data Figure1.pdf]

# Isobutyrate

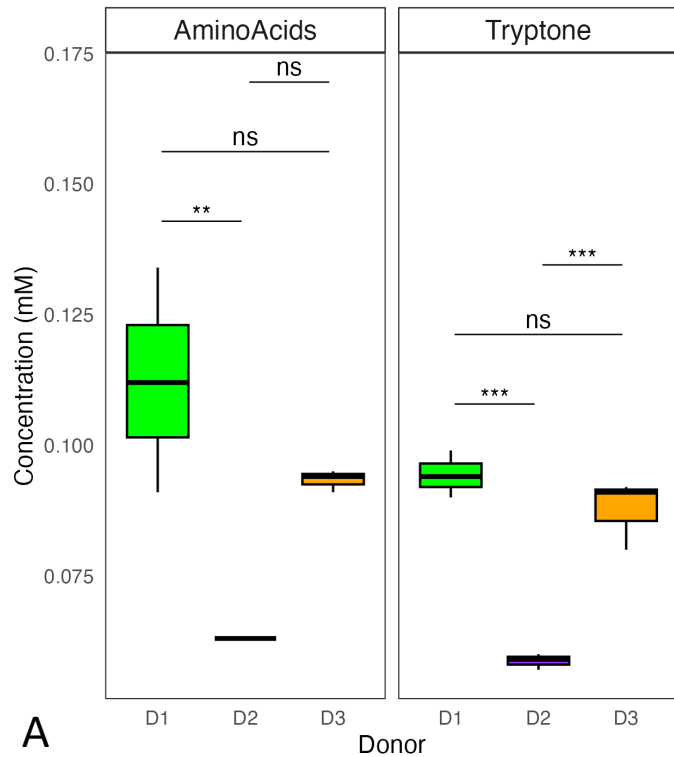

# Isovalerate

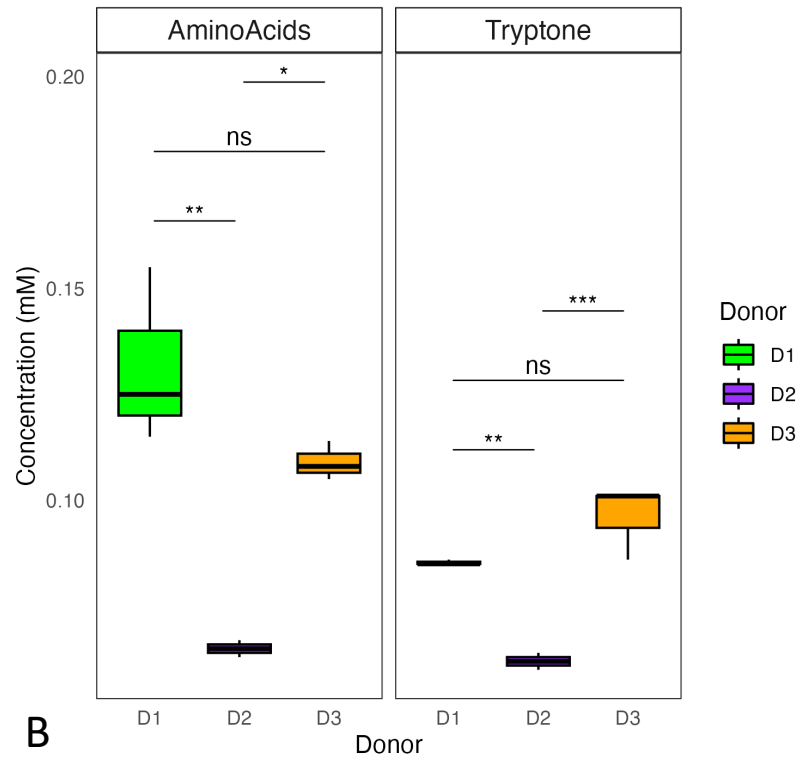

Supplement: fiae078_Supplemental_Files [file fiae078_supplemental_files.zip › Supp data Figure2.pdf]

**Community Stability Donor 1**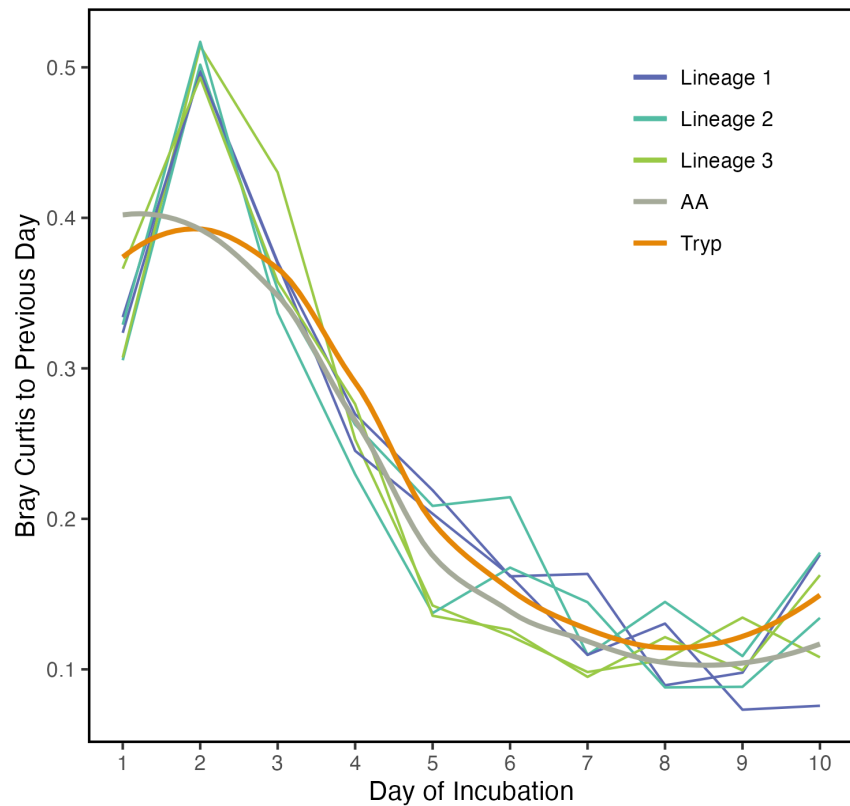**Community Stability Donor 2**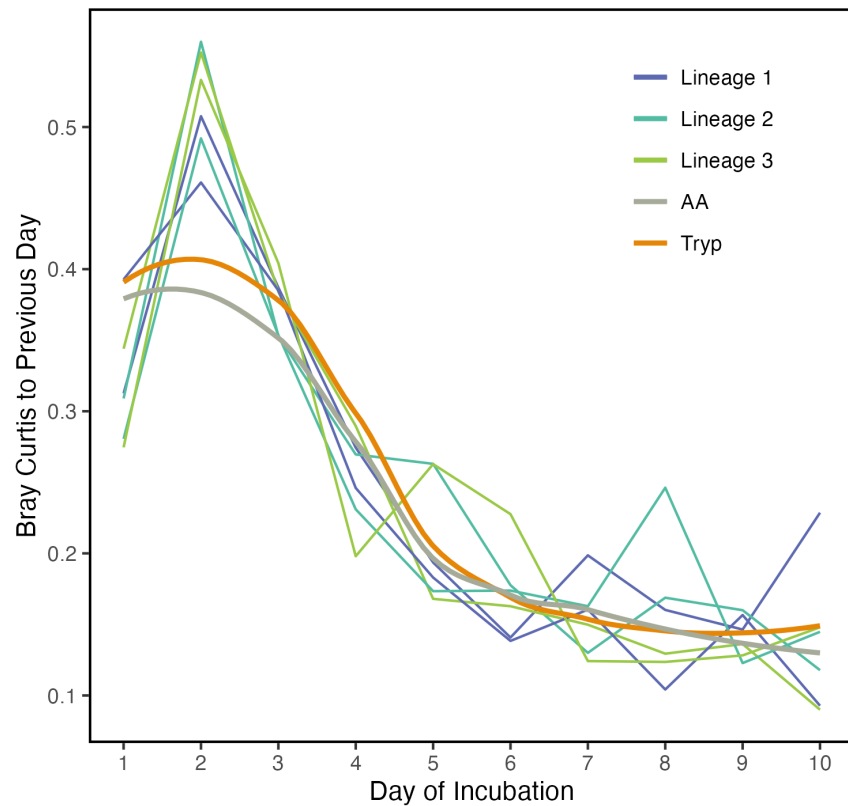**Community Stability Donor 3**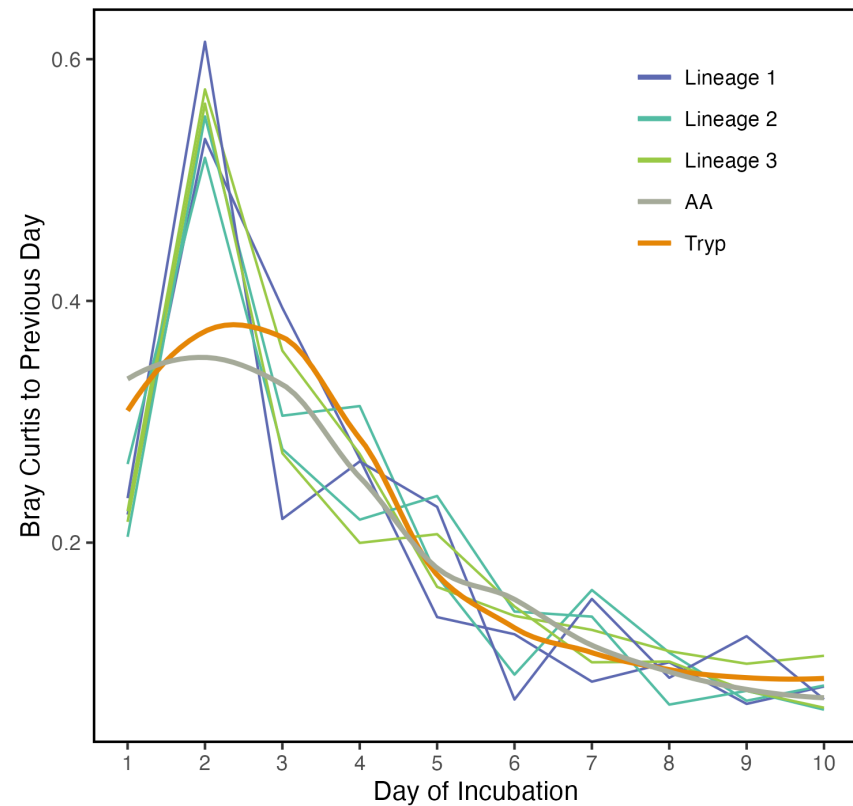

Supplement: fiae078_Supplemental_Files [file fiae078_supplemental_files.zip › Supp data Figure3.pdf]

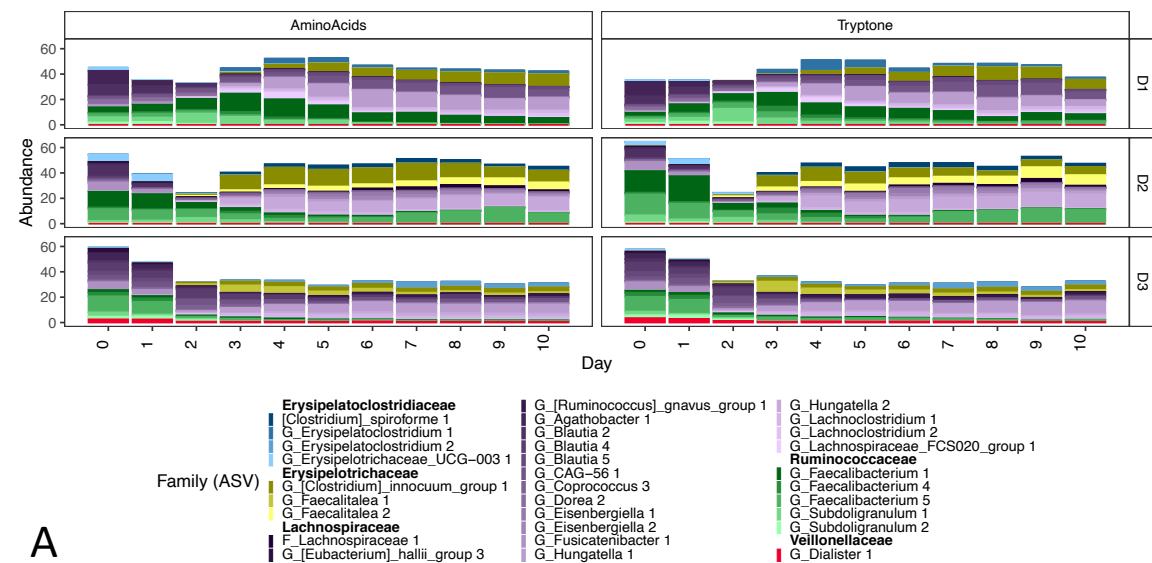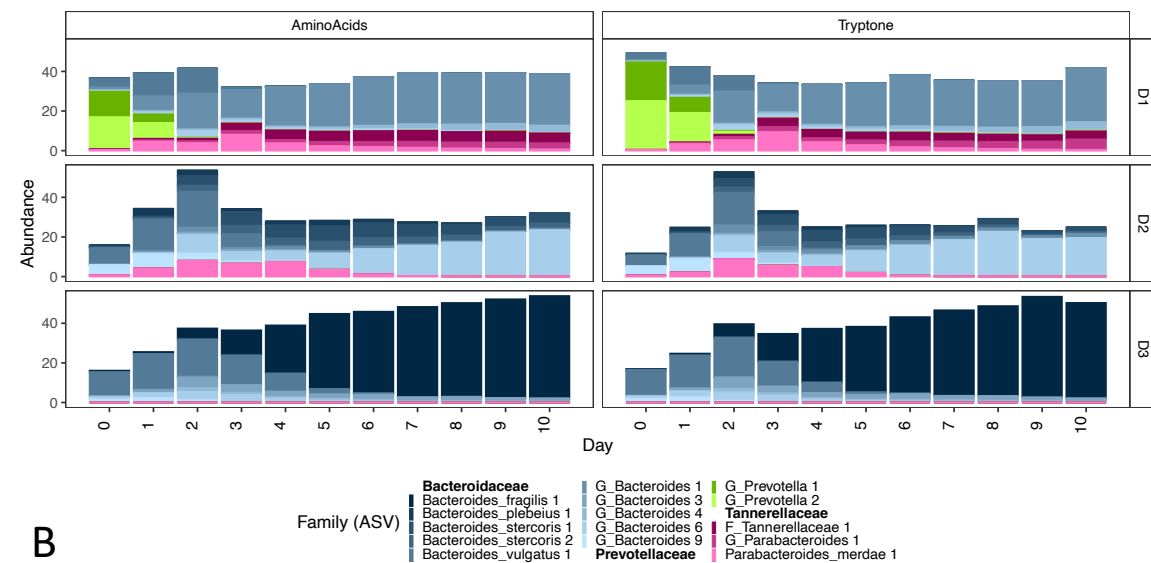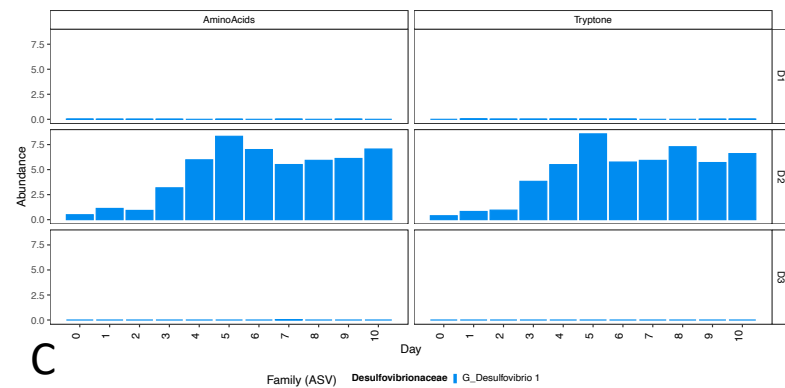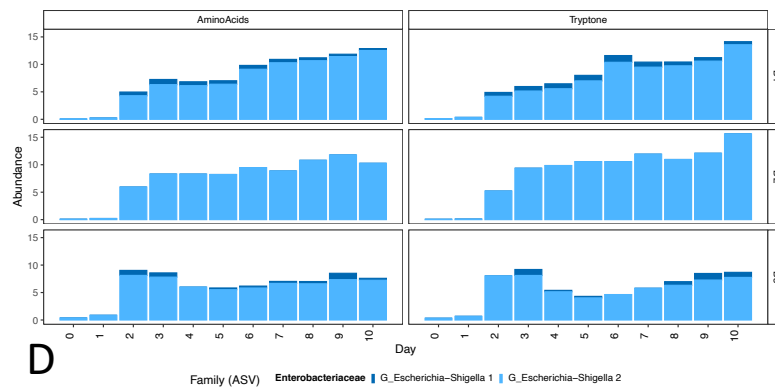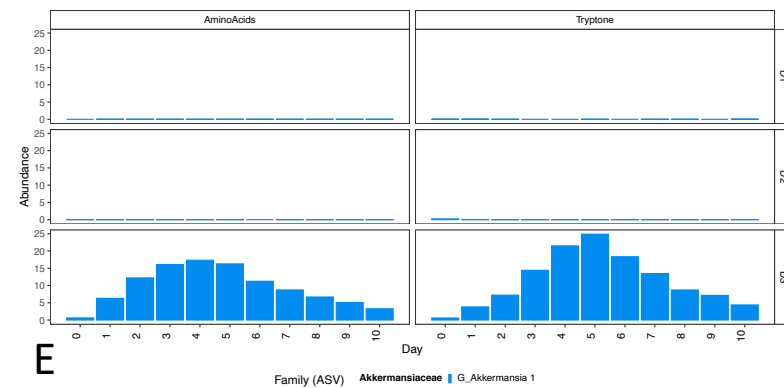

Supplement: fiae078_Supplemental_Files [file fiae078_supplemental_files.zip › Supp data Figure5.pdf]

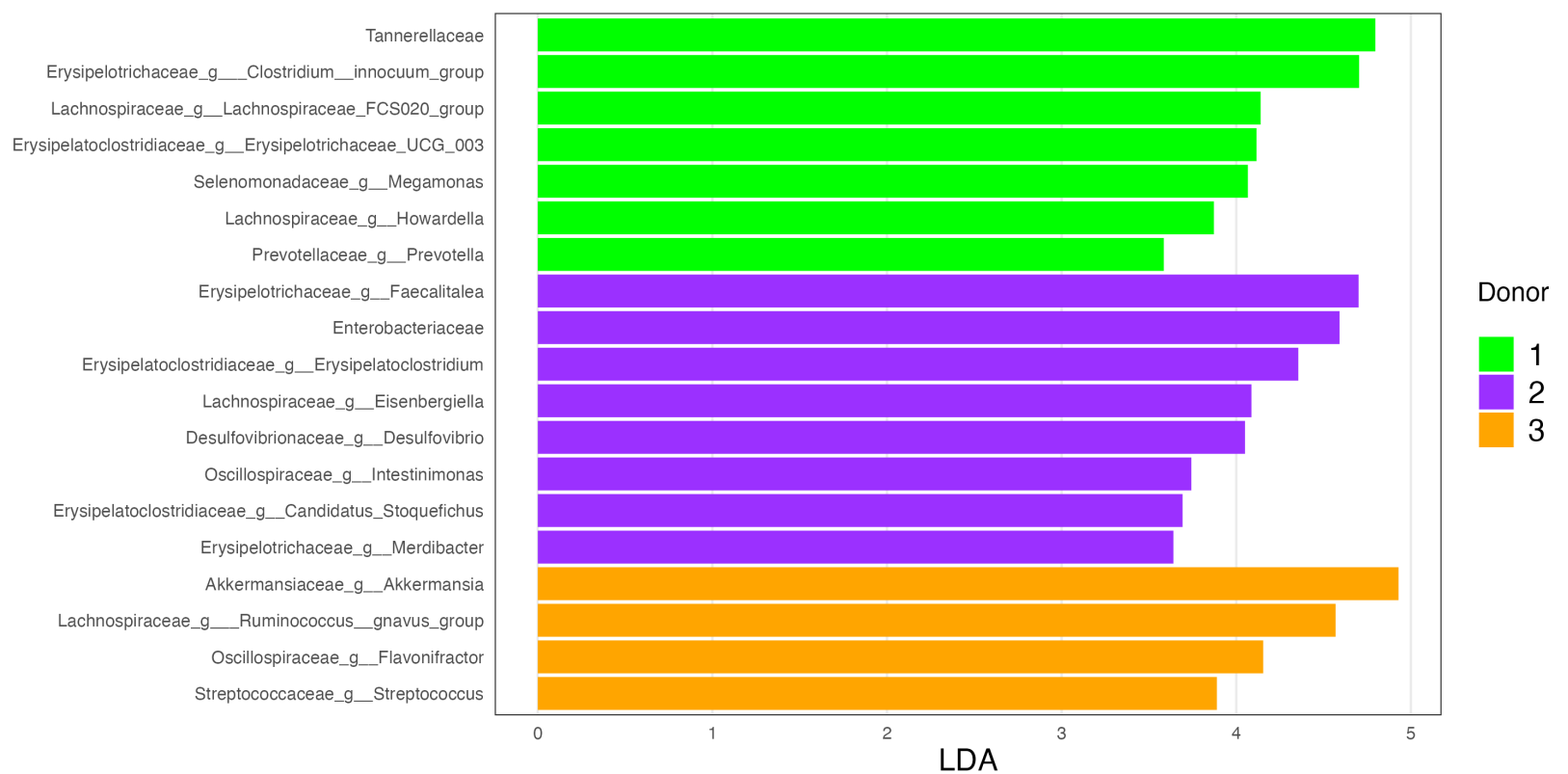

Supplement: fiae078_Supplemental_Files [file fiae078_supplemental_files.zip › Supp data Figure6.pdf]
